# Tumor-specific mutations in low-frequency genes affect their functional properties

**DOI:** 10.1007/s11060-015-1741-1

**Published:** 2015-02-19

**Authors:** Lale Erdem-Eraslan, Daphne Heijsman, Maurice de Wit, Andreas Kremer, Andrea Sacchetti, Peter J. van der Spek, Peter A. E. Sillevis Smitt, Pim J. French

**Affiliations:** 1 Department of Neurology, Be 430A, Erasmus Medical Center, PO Box 2040, 3000 CA Rotterdam, The Netherlands; 2 Department of Bioinformatics, Erasmus Medical Center, Rotterdam, The Netherlands; 3 Department of Pathology, Josephine Nefkens Institute, Erasmus Medical Center, Rotterdam, The Netherlands

**Keywords:** Oligodendroglioma, Infrequent mutations, Sequencing

## Abstract

Causal genetic changes in oligodendrogliomas (OD) with 1p/19q co-deletion include mutations in *IDH1, IDH2, CIC*, *FUBP1, TERT* promoter and *NOTCH1*. However, it is generally assumed that more somatic mutations are required for tumorigenesis. This study aimed to establish whether genes mutated at low frequency can be involved in OD initiation and/or progression. We performed whole-genome sequencing on three anaplastic ODs with 1p/19q co-deletion. To estimate mutation frequency, we performed targeted resequencing on an additional 39 ODs. Whole-genome sequencing identified a total of 55 coding mutations (range 8–32 mutations per tumor), including known abnormalities in *IDH1*, *IDH2*, *CIC* and *FUBP1*. We also identified mutations in genes, most of which were previously not implicated in ODs. Targeted resequencing on 39 additional ODs confirmed that these genes are mutated at low frequency. Most of the mutations identified were predicted to have a deleterious functional effect. Functional analysis on a subset of these genes (e.g. *NTN4* and *MAGEH1*) showed that the mutation affects the subcellular localization of the protein (n = 2/12). In addition, HOG cells stably expressing mutant *GDI1* or *XPO7* showed altered cell proliferation compared to those expressing wildtype constructs. Similarly, HOG cells expressing mutant *SASH3* or *GDI1* showed altered migration. The significantly higher rate of predicted deleterious mutations, the changes in subcellular localization and the effects on proliferation and/or migration indicate that many of these genes functionally may contribute to gliomagenesis and/or progression. These low-frequency genes and their affected pathways may provide new treatment targets for this tumor type.

**Electronic supplementary material:**

The online version of this article (doi:10.1007/s11060-015-1741-1) contains supplementary material, which is available to authorized users.

## Introduction

Oligodendrogliomas (ODs) account for 20 % of all glial tumors and are thought to arise from oligodendroglial precursor cells (OPCs). They are classified as either grade II or grade III and have a more favorable clinical and prognostic outcome with respect to other gliomas [1].

Frequently occurring driver mutations in oligodendrogliomas include mutations in *IDH1*, *CIC*, *FUBP1, TERT* promoter and *NOTCH* [2–6]. However, tumor formation is assumed to require more somatic mutations [7]. Interestingly, many other somatic mutations within protein-coding genes have also been identified in oligodendrogliomas, albeit at a low frequency [8]. The role of most of these infrequent mutations in tumorigenesis and/or progression is unclear. To date, only few studies have suggested a functional impact of genes mutated at low frequency in gliomas [9–13]. Therefore, low-frequency genes that play a role in the initiation and/or progression of ODs need to be identified to better understand OD pathogenesis and for development of targeted therapies.

Here, we have performed whole-genome sequencing on three ODs to identify all genetic changes in these tumors. We then performed targeted resequencing on an additional 39 tumors to demonstrate that many of these mutations occur at low frequency. Functional analysis of a subset of these low-frequency genes (*NTN4*, *GDI1*, *MAGEH1*, *SASH3*, *ZNF238*, *OR5D14*, *ZNF57*, *DCUN1D2*, *ARSE*, *XPO7*, *GABRE* and *PGLYRP4*) suggest that they can contribute to tumor pathogenesis and therefore are unlikely to be passengers. These genes and their affected pathways open up entirely novel treatment targets for this tumor type.

## Materials and methods

### Patient material

OD samples were collected from the Erasmus MC tumor archive. Use of patient material was approved by the Institutional Review Board and patients provided written informed consent according to national and local regulations for the clinical study and correlative tissue studies. After surgical resection, all samples were snap-frozen and stored at −80 °C. Non-neoplastic DNA was isolated from blood and stored at −80. Assessment of 1p19q LOH was performed previously [14, 15]. Patient characteristics are listed in supplementary Table 1.

### DNA extraction and sequencing

Tumor DNA was extracted from fresh frozen (FF) tumor samples using the AllPrep DNA/RNA mini kit (Qiagen, Venlo, the Netherlands). Matched normal DNA was isolated from 1 ml blood as part of routine diagnostic procedures. Whole-genome sequencing was performed by Complete Genomics (Mountain View, US) using 5 µg DNA. Whole-genome sequencing data analysis was performed using cgatools version 1.4.0 and described in supplementary methods. All mutations were validated by Sanger sequencing. Targeted resequencing on an additional 39 ODs was performed by Baseclear (Leiden, the Netherlands). Library construction and resequencing data analysis were described in supplementary methods.

### Cell lines and sorting

Constructs of wildtype and mutated genes were generated by site directed mutagenesis (n = 12), and C-terminally fused to GFP for visualization. Subsequently, Human oligodendroglial (HOG) cells [16] and HEK 293 cells were transiently transfected with wildtype or mutant constructs using lipofectamine according to the manufacturer’s instructions. Stably transfected HOG cells were created as described in supplementary methods.

### Functional analysis

For proliferation experiments, cells (50.000 cells/well) were plated in a 24-well Greiner plate (Greiner Bio-One, Alphen a/d Rijn, the Netherlands) and followed using an Incucyte (Essenbioscience, Hertfordshire, United Kingdom). Growth curves were constructed using the Confluence v1.5 metric of the Incucyte software.

For migration experiments, cells were grown to confluence in a 24-well Essen ImageLockplate after which a cell-free zone (scratch) was created using a WoundMaker. Wells were then washed in PBS after which serum-free media was added to the plates. All plates were followed for 5 days and images were automatically captured at 3-h intervals from 3 to 4 separate regions within a well. Relative wound density, wound width and wound confluency curves were constructed using data points of every capture. All proliferation and migration experiments were performed at least in triplicate.

Flow cytometric cell cycle analysis using propidium iodide was performed as described in supplementary methods.

## Results

### Whole-genome sequencing

**Fig. 1:**
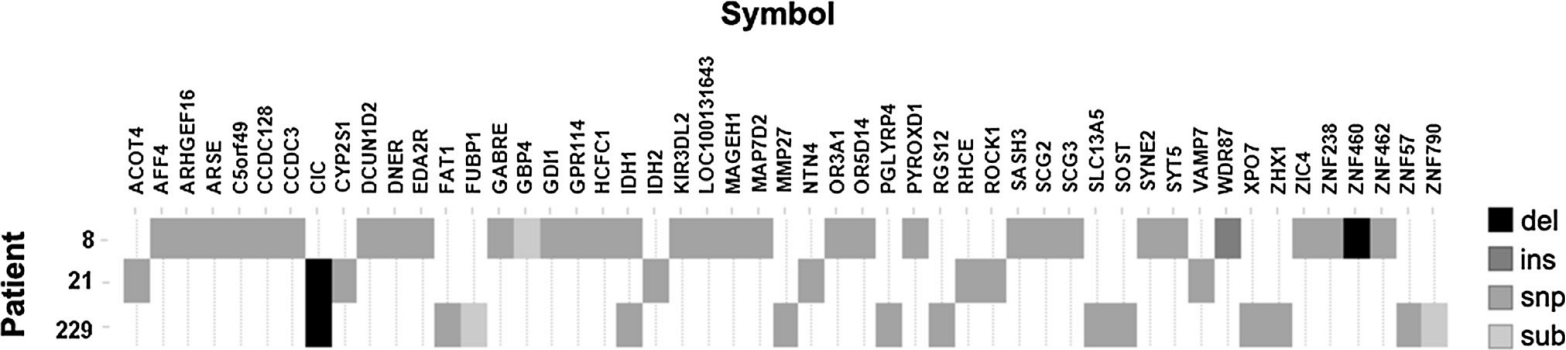
Somatic genetic alterations in three oligodendrogliomas identified by whole-genome sequencing. Shown are all mutations identified in three tumors that (i) were localized to the coding regions of exons,( ii) were nonsynonymous, (iii) were absent from dbSNP130 and (iv) had a somatic score >−20. Alterations are coded in greyscale by the type of mutation (deletion, insertion, snp, substitution). The number of alterations range from 8 to 32 variants per sample. All 55 mutations were validated by Sanger sequencing

To identify somatic alterations in oligodendrogliomas, we performed whole-genome sequencing on DNA of three ODs and their matched germline DNA. All tumors were WHO grade III and had 1p19q co-deletion. The mapped sequence of the six samples varied between 237 and 249 Gb, resulting in a coverage between respectively 83 and 90-fold per genome. Confident diploid calls could be made for 94–95 % of the reference genome. On average 3,5 million genetic variants were identified per sample. Of these, 55 (range of 8–32 variants per sample), were localized to coding exons, were neither synonymous nor present in dbSNP130 and had a somatic score >−20 (see supplementary methods) (Fig. 1). Identified variants consisted of missense (84 %), nonsense (9 %) and frameshifts (7 %). All 55 mutations were validated by Sanger sequencing (supplementary Table 2).

We performed an in silico analysis on all 55 genes to estimate the effect of mutation on protein. Polyphen-2 predictions were available for 43 genes of which 22 were probably damaging, 9 possible damaging, and 12 were benign. In randomly picked mutations identified with a lower confidence score (i.e. somatic scores ≤−20) the rate of damaging mutations was significantly lower: 10 probably damaging, 8 possible damaging and 21 benign changes, (p = 0.033, Chi square test). The identified mutations also had a slight tendency towards a higher conservation compared to mutations with somatic scores ≤−20: GERP-score 2.71 ± 2.96 v 1.75 ± 3.61 p = 0.156). The 55 mutations identified by whole-genome sequencing therefore were often predicted to have deleterious effects on the protein.

### Targeted resequencing

Because we aimed to determine functional effects of genes mutated at low frequency in gliomas, we performed targeted resequencing of 44 (of the 55 identified by whole-genome sequencing) genes that were thusfar not implicated in gliomagenesis (supplementary Table 2). Resequencing was performed on the entire coding region of the 44 genes on 39 grade II/III ODs of which 28/39 had 1p/19q LOH, 5/39 had loss of 1p or 19q and 6/39 had no 1p19q loss. We chose this dataset because it represents a typical cohort of histologically diagnosed oligodendrogliomas where most will have 1p19q co-deletion, but some have other, more aggressive genetic changes.

**Fig. 2:**
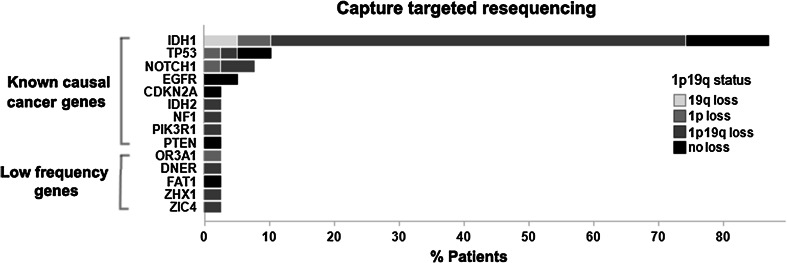
Targeted resequencing on 39 ODs confirms that many genes are mutated at low frequency. Of the known cancer genes (*IDH1/2, TP53, NOTCH1, EGFR, CDKN2A, NF1, PIK3R1 and PTEN*), a total of 48 mutations (range 1–4 mutations per tumor) were identified in 39 tumors (known causal cancer genes). Of the genes identified by whole-genome sequencing that thus far are not implicated in oligodendrogliomas, no mutations were found in 39 of the 44 genes in any of the additional 39 tumors (not shown). In the remaining five genes, we identified mutations in only one additional sample (indicated in the figure as low-frequency genes). Distribution of 1p19q status is reported for each identified mutation. Mutations in *EGFR*, *CDKN2A* and *PTEN* were only found in patients with intact 1p19q status

No mutations were found in 39 of the 44 genes in any of the additional 39 tumors. Of the remaining five genes, mutations were identified in only one additional sample (Fig. 2, supplementary Table 4). In our samples, the mutations were all present at a high allele frequency (range 39–84 %) suggesting that the mutation is present in a large proportion of the resected tumor, at least within the tissue investigated. Among the 28 tumors with 1p19q co-deletion, mutations in only three of the 44 genes were identified (supplementary Table 5). Although in individual tumors the allele frequency was high, the overall mutation frequency of all 44 genes was low. The mutations in the 44 genes identified by our screen therefore are ‘low-frequency genes’: genes mutated at low frequency in oligodendrogliomas.

Apart from these low-frequency genes, we included a set of known cancer genes (*IDH1, IDH2, PTEN, TP53, NOTCH1, EGFR, CDKN2A, CDKN2B, NF1* and *PIK3R1*) for reference on our targeted resequencing effort. Analysis of these known cancer-relevant genes revealed a total of 48 mutations (range of 1–4 mutations per tumor), which consisted of missense (44) and nonsense (4) variants. These include mutations in *IDH1* (34 mutations in 39 tumors), *TP53* (4/39), *NOTCH1* (3/39), *EGFR* (2/39), *IDH2* (1/39), *CDKN2A* (1/39), *NF1* (1/39), *PIK3R1* (1/39) and *PTEN* (1/39) (Fig. 2, supplementary Table 4). Mutations in *EGFR*, *CDKN2A* and *PTEN* were only found in patients with intact 1p19q chromosomes. Targeted resequencing therefore confirmed a high frequency of mutations in genes that drive oligodendrogliomas.

To better determine the incidence of the identified mutations, we analyzed exome sequencing data of 7 oligodendrogliomas from Bettegowda et al. [2], 16 oligodendrogliomas from Yip et al. [5] and 170 low-grade gliomas (LGG) and 290 glioblastomas (GBM) [8] from the TCGA dataset. In this large dataset, most [36/44] mutations identified by whole-genome sequencing that thus far are not implicated in oligodendrogliomas, were also identified in one of these datasets; eight were uniquely identified by us. The frequency was however low: often only one sample was identified in the external datasets with a mutation in that gene (supplementary Fig. 1).

### Mutations in low-frequency genes can affect the proteins’ subcellular localization

**Fig. 3:**
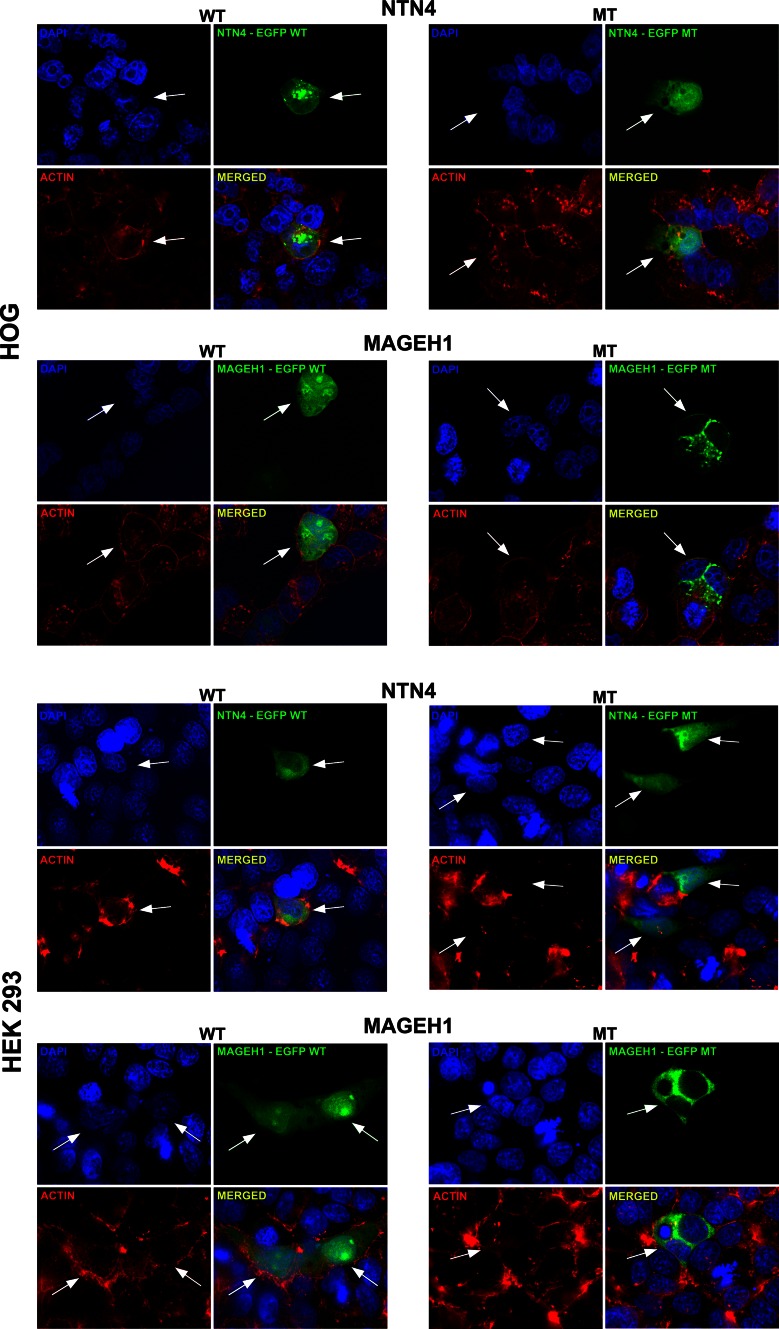
Mutations in low-frequency genes can affect the subcellular localization of proteins. HOG and HEK 293 cells were transiently transfected with either the wildtype or mutant construct and stained for GFP (*green*), DAPI (*blue*) and Alexa fluor phalloidin (*red*). A total of twelve wildtype and mutant construct pairs were made. In both cell lines, the mutated *NTN4* construct showed a stronger nuclear staining pattern compared to the wildtype construct. For *MAGEH1*, the mutation resulted in a reduced or even absent nuclear staining. No differences in the subcellular localization between wildtype and mutant constructs were found in HOG and HEK 293 cells expressing *GDI1, ZNF238, SASH3, XPO7, ZNF57, GABRE, OR5D14, PGLYRP4*, *ARSE* and *DCUN1D2* (not shown)

**Fig. 4:**
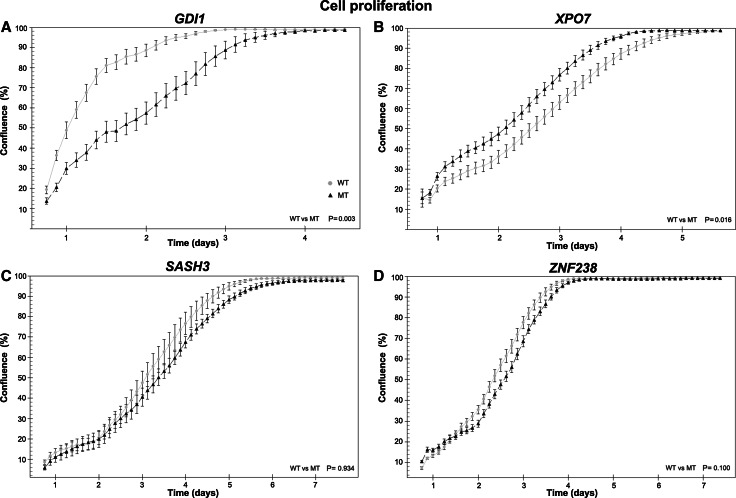
Mutations in low-frequency genes can affect cell growth. **a** HOG cells stably expressing wildtype *GDI1* show increased proliferation compared to *GDI1* ^R193C^ expressing cells (p = 0.003, n = 2 independent experiments). **b** Cells stably expressing *XPO7* ^D237N^ show a higher rate of proliferation during the first 24 h, compared to wildtype expressing cells (p = 0.02, (n = 2)). After 24 h, similar proliferation rates were observed in wildtype and *XPO7* ^D237N^. **c** HOG cells stably expressing *SASH3* wildtype or *SASH3* ^R288*^ and **d** wildtype or *ZNF238* ^V121I^ show similar proliferation rates (n = 3)

**Fig. 5:**
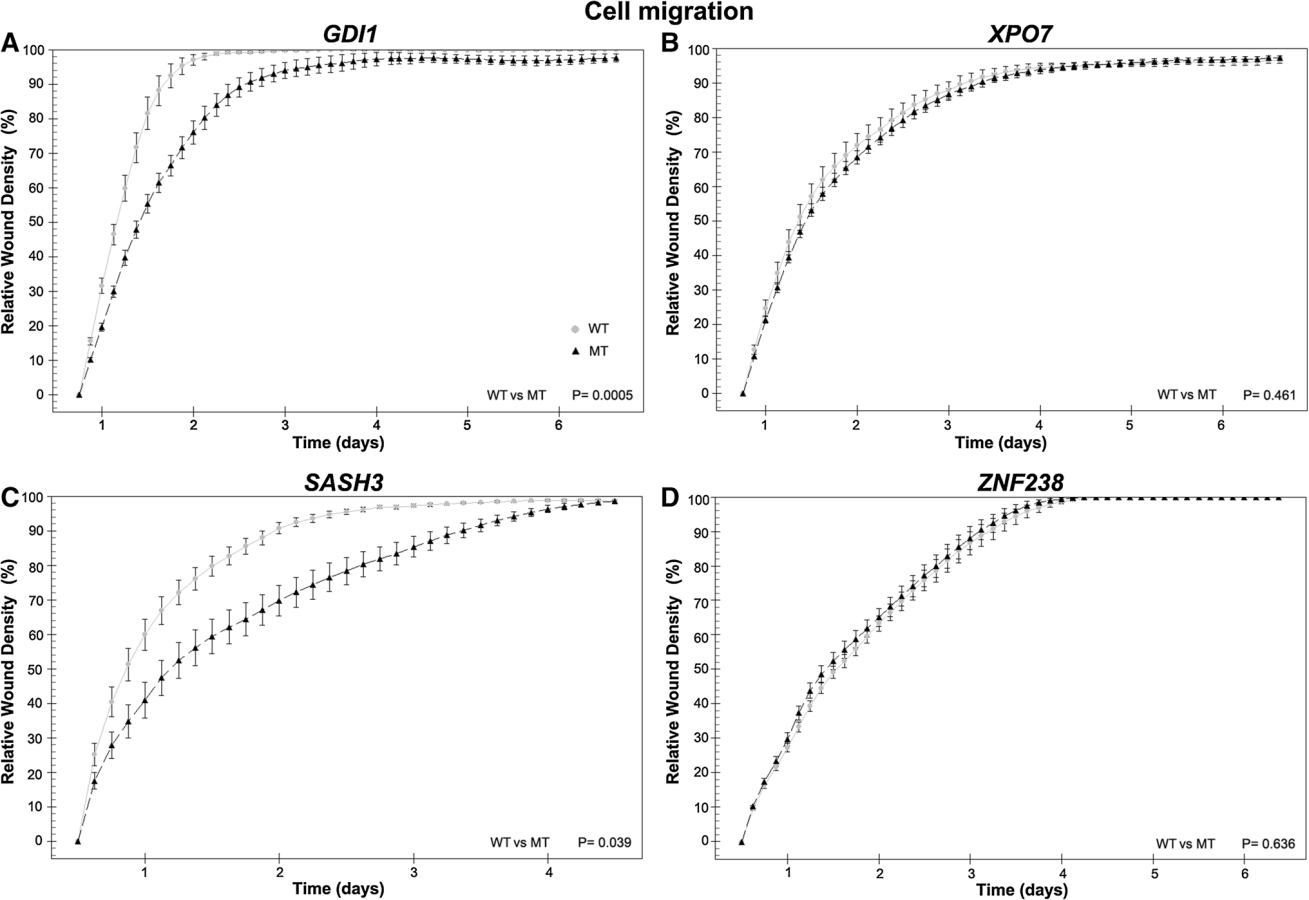
Mutations in low-frequency genes can affect cell migration. **a** HOG cells stably expressing *GDI1* wildtype have a higher migration rate compared to *GDI1* ^R193C^ expressing cells (p = 0.003, n = 2 independent experiments). **b** No differences in migration were observed between cells expressing wildtype and *XPO7* ^D237N^ or between cells expressing *ZNF238* wildtype and mutant (**d**) (p = 0.5). **c** Cells expressing wildtype *SASH3*, show increased migration compared to *SASH3* ^R288*^(p = 0.039)

In order to determine whether these low-frequency genes may be involved in glioma pathogenesis, we performed a more detailed molecular analysis on 12/44 of those genes (Figs. 3, 4, 5). These 12 genes were randomly picked from the 44 low-frequency genes. Transient transfection revealed that in 2/12 constructs, the mutation affects the proteins’ subcellular localization. In the gene *NTN4*, the mutation resulted in a strong nuclear localization, which was absent in the wildtype (Fig. 3). For *MAGEH1*, the mutation resulted in a reduced or even absent nuclear localization. No differences in the subcellular localization between wildtype and mutant constructs were found in cells expressing *GDI1*, *ZNF238*, *SASH3*, *XPO7*, *ZNF57*, *GABRE,* *OR5D14, PGLYRP4*, *ARSE* and *DCUN1D2* (not shown).

### Low-frequency genes can affect proliferation and/or migration

To further examine the functional properties of low-frequency genes, we generated cell lines stably expressing the mutant or wildtype variant of four genes and performed functional analysis on proliferation and migration (Figs. 4, 5).

The first gene examined was *GDI1*, in which the mutation was located in the geranyl–geranyl transferase domain (c.577C > T, p.R193C). HOG cells stably expressing wildtype *GDI1* have increased proliferation compared to those expressing *GDI1* ^R193C^ or eGFP (p = 0.003, p = 0.04, respectively, n = 2). This is also confirmed by flow cytometry, showing that in cells expressing wildtype *GDI1*, 7.5 % more cells are found in the S-G2-M phase and 8.5 % less in the G1 compared to those expressing *GDI1* ^R193C^ (supplementary Fig. 2). No differences in proliferation were observed between *GDI1* ^R193C^ and eGFP cells (p = 0.7). Similarly, HOG cells stably expressing *GDI1* wildtype showed increased migration compared to *GDI1* ^R193C^ or eGFP (p = 0.003, p = 0.0005 respectively, n = 2). No differences in migration were observed between *GDI1* ^R193C^ and eGFP cells (p = 0.3). The differences in both proliferation and migration between wildtype and mutant constructs indicate that the mutation in *GDI1* affects the functional property of the protein.

Our second gene examined was *XPO7*, in which a mutation was identified in the ARM-type fold domain (c.709G > A, p.D237N). An increase in proliferation was observed during the first 24 h in cells stably expressing *XPO7* ^D237N^ compared to wildtype and eGFP cells (p = 0.02, p < 0.001 (n = 2), respectively). After 24 h, the proliferation rate was similar in cells stably expressing wildtype, *XPO7* ^D237N^ and eGFP. Because of the differences in proliferation at the start of the experiment, *XPO7* ^D237N^ cells reach confluency more rapid than wildtype or eGFP cells. The initial difference was consistently observed in multiple experiments with 3–6 wells per experiment and 4 positions per well. Because cells were plated at 2 different densities (50.000 and 100.000 cells per well) the observed difference in initial proliferation rates appeared independent of plating density. A possible explanation for this difference is that *XPO7* ^D237N^ cells recover more rapidly after plating. Migration of cells expressing *XPO7* ^D237N^ or wildtype was higher than those expressing eGFP (p = 0.05, p = 0.03, respectively (n = 2)). No differences in migration were observed between cells expressing *XPO7* ^D237N^ and wildtype (p = 0.5). The effect of the identified point mutation in *XPO7* on proliferation highlights its importance for protein function in these cells.

The third gene examined was *SASH3*, in which the mutation was located in the SAM domain (c.862C > T), partially disrupting this domain in the C-terminal region (p.R288*). HOG cells stably expressing *SASH3* wildtype, *SASH3* ^R288*^ or eGFP have a similar proliferation rate (n = 3). However, cells expressing wildtype *SASH3,* show increased migration compared to *SASH3* ^R288*^ or eGFP. The difference in migration between wildtype and *SASH3* ^R288*^indicates that the mutation affects the function of the wildtype protein (p = 0.001, n = 2).

Our last gene examined was *ZNF238*, in which the mutation was located in a BTB/POZ fold domain (c.361G > A, p.V121I). In HOG cells stably expressing *ZNF238* wildtype or *ZNF238* ^V121I^ constructs, we observed a slight decrease in proliferation compared to eGFP expressing cells (p < 0.001, n = 4). A more pronounced effect was observed in migration: HOG cells stably expressing *ZNF238* wildtype and *ZNF238* ^V121I^ show a strong decrease compared to eGFP. These results were consistently observed in multiple experiments (p < 0.001, n = 4). Although no differences were observed between wildtype and mutant constructs, the results do indicate that wildtype or *ZNF238* ^V121I^ constructs affect cellular proliferation and migration.

## Discussion

In this study, we have performed whole-genome and targeted resequencing on 3 and 39 oligodendrogliomas to identify somatic mutations. Apart from the known frequently mutated genes such as *IDH1*, *CIC* and *FUBP1*, our study also identified genes that were infrequently mutated (i.e. in one or two samples only). Most low-frequency genes are predicted to affect the protein’s function by in silico analysis. A significantly higher proportion of genes had a potential deleterious effect compared to mutations in genes identified with a lower confidence score (i.e. somatic scores ≤−20). Functional analysis of these low-frequency genes indicated that the identified mutation can affect protein subcellular localization and/or cell physiology. Our results therefore suggest that (at least some of) these genes may be relevant for gliomagenesis and/or contribute to progression.

The frequency of mutations identified at a higher frequency in our study (*IDH1*, *CIC* and *FUBP1*) are similar to those reported by others for this tumor type. Mutations in the *ATRX* gene were not identified; this gene is frequently mutated in other glioma subtypes including WHO grade II-III astrocytomas (71 %), oligoastrocytomas (68 %) and secondary glioblastomas (57 %) [2, 5]. Also similar to reported by others, mutations in *EGFR*, *CDKN2A* and *PTEN* were mutually exclusive with 1p19q co-deletion [2–5, 8].

To our knowledge, our study is the first to functionally study low-frequency genes on a larger scale. Several other studies have demonstrated a functional impact of genes mutated at a low frequency [9–13, 17]. Our data therefore are in line with the hypothesis that genes mutated at low frequency in gliomas can functionally contribute to gliomagenesis.

Of the genes examined, the mutations identified in *NTN4* and *MAGEH1* were predicted as “probably damaging” by Polyphen-2 analysis and affected the protein subcellular localization. Both mutations in *NTN4* and *MAGEH1* are not located in any of the known signal peptides for protein localization . These mutations may affect protein folding and sorting, however this would be accompanied by an accumulation of proteins in the ER, and we did not observe such accumulation in our transfected cells.

Of the genes examined in more detail, the mutation identified in *GDI1* was predicted as “probably damaging” by Polyphen-2 analysis and did not affect the protein subcellular localization. Our data show that HOG cells stably expressing wildtype *GDI1* have increased proliferation and migration compared to those expressing *GDI1* ^R193C^ or eGFP. The proliferation experiments were validated by flow cytometry, showing that wildtype *GDI1* expressing cells are more present in the S-G2-M phase and less in the G1 compared to mutant *GDI1* expressing cells. *GDI1* encodes for GDP dissociation inhibitor 1, which is involved in recycling of Rab proteins and contains a GTPase activation GDI1-β2/GDI1-β and geranyl–geranyl transferase domain (supplementary Fig. 3) [18–20]. The GTPase activating domain of *GDI1* interacts with the GDP-bound Rab proteins, while the geranyl–geranyl domain interacts with the prenylated binding motif of Rab protein [21].

The altered migration may be caused by a differential activation of Rab proteins by *GDI*. Rab proteins belong to the Ras superfamily and are involved in vesicle trafficking between cellular compartments along actin or microtubules [22]. Binding of *GDI* to prenylated GDP-bound Rab protein in the cytosol mediates the delivery of Rab proteins to membranes during vesicle formation and their return into the cytosol after vesicle fusion [23, 24]. Importantly, Rab proteins seem to direct migration of cancer cells by regulating integrin recycling [25]. This mutation is functionally important as mutated *GDI1* does not stimulate cell proliferation and migration whereas wildtype *GDI1* does.

The second gene examined was *XPO7*, in which the mutation was also predicted to be probably damaging by Polyphen-2 analysis. The mutation did not affect the protein subcellular localization. Our data indicate that both wildtype and *XPO7* ^D237N^ increase the proliferation rate of HOG cells. *XPO7* encodes for exportin 7, which is a nuclear transport receptor that exports cargos from the nucleus into the cytoplasm [26]. This protein contains a N-terminal Importin-beta domain that binds to RAN and an ARM-type fold domain [26]. Its C-terminal region is thought to be involved in the recognition of substrates with broad specificity [27]. The fact that both wildtype and *XPO7* ^D237N^ stimulate cell proliferation and migration, highlights the importance of this gene.

The third gene examined was *SASH3,* in which the mutation was predicted as probably damaging by Polyphen-2 analysis and did not affect the protein subcellular localization. The current study found that cells expressing wildtype *SASH3,* but not *SASH3* ^R288*^, show increased migration, indicating that the mutation affects the function of the wildtype protein. *SASH3* encodes a signaling adapter protein, containing a SLY motif in the N-terminal region, a SH3 motif and a SAM motif in the C-terminal region [28]. SAM families of receptors are known to play a role in many developmental processes including cell migration, neuronal formation and angiogenesis [28]. They seem to mediate signal transduction by connecting downstream effector proteins to cell surface receptors [29, 30]. The altered migration pattern in *SASH3* ^R288*^ expressing cells may therefore be caused by disturbed signal transduction pathways that lead to cell migration. This mutation is functionally important as *SASH3* ^R288*^ does not stimulate cell migration where wildtype *SASH3* does.

The last gene examined was *ZNF238,* in which the mutation was predicted as “probably damaging” by Polyphen-2 analysis and did not affect the protein subcellular localization. Our data indicate that cells stably expressing wildtype or *ZNF238* ^V121I^ decrease proliferation and migration compared to control. The present finding is consistent with other studies in which *ZNF238* was found to decrease proliferation [31, 32] and that *ZNF238* is essential for neuronal migration in experimental mouse models [32–34]. *ZNF238* encodes a transcriptional repressor that contains a BTB/POZ fold domain in the N-terminal region and four zinc fingers in the C-terminal region [35, 36]. This protein family plays a role in many processes, including DNA damage response, cell cycle and developmental processes [31–34, 37]. Our observation that wildtype and mutant constructs affect cellular proliferation and migration, highlights the important role of this gene.

Although our data argue that many genes are functionally relevant for gliomas, there are some limitations to our study. For all functional experiments, we have only utilized the HOG cell line, as these cells have been well characterized and resemble immature oligodendrocytes [16]. Whether these effects are retained in other cell lines remains to be determined. For example, to determine whether these genes indeed contribute to gliomagenesis, similar experiments need to be performed in stem cells, patient derived xenografts and various in vivo experiments. However because mutations occur at such low frequency, patient-derived primary cultured tumor cell lines containing these mutations are hard to obtain. Moreover, oligodendrogliomas with 1p19qLOH and/or *IDH1* mutated tumors virtually cannot be propagated in vitro. Nevertheless, our data shows that even in a single cell line, a substantial proportion of low-frequency genes functionally affects cell physiology.

Another limitation of this study is that we have only performed functional analysis of a few genes. However, in a substantial proportion of the examined genes, the mutation affected the function of the wildtype protein. Our data therefore suggest that a substantial proportion of low-frequency genes are functionally relevant for glioma initiation and/or progression.

In contrast to may be expected for oncogenic mutations, we found that the proliferation and/or migration rate was reduced in cells expressing *GDI1* ^R1193C,^ *ZNF238* ^V121I^ and *SASH3* ^R288*^. It is not uncommon for ectopic expression of oncogenes in various cell lines to result in reduced proliferation and altered migration. In fact, Sun et al. have shown that expression of *EGFR* in mutant melanoma cells confers a growth disadvantage that is further strengthened by the addition of *EGFR* ligand [38]. A similar growth disadvantage (and altered migration pattern) was observed when expressing *IDH1* ^R132H^ [10, 39]. Perhaps, this is a relatively common physiological response of cells that have never been dependent on a specific oncogene. It is however also possible that the mutation does not contribute to tumor formation/progression but simply has deleterious effects on the functioning of the wildtype protein.

In conclusion, we have demonstrated that low-frequency genes can affect the proteins’ subcellular localization and/or physiology in HOG cells. These findings indicate that low-frequency genes functionally can contribute to gliomagenesis and/or progression and suggest these genes as new therapeutic targets for treatment for this tumor type.

## Electronic supplementary material

Below is the link to the electronic supplementary material.

Supplementary material 1 (PNG 38 kb)

Supplementary material 2 (PNG 31 kb)

Supplementary material 3 (PNG 86 kb)

Supplementary material 4 (DOCX 18 kb)

Supplementary material 5 (DOC 34 kb)

Supplementary material 6 (DOC 76 kb)

Supplementary material 7 (DOC 113 kb)

Supplementary material 8 (DOC 829 kb)

Supplementary material 9 (DOC 109 kb)

Supplementary material 10 (DOC 92 kb)
