# Supplementary figures and images for: Tumor-specific mutations in low-frequency genes affect their functional properties
Source: J Neurooncol. 2015 Feb 19;122(3):461–70. doi: 10.1007/s11060-015-1741-1 (PMC4436689; doi:10.1007/s11060-015-1741-1)

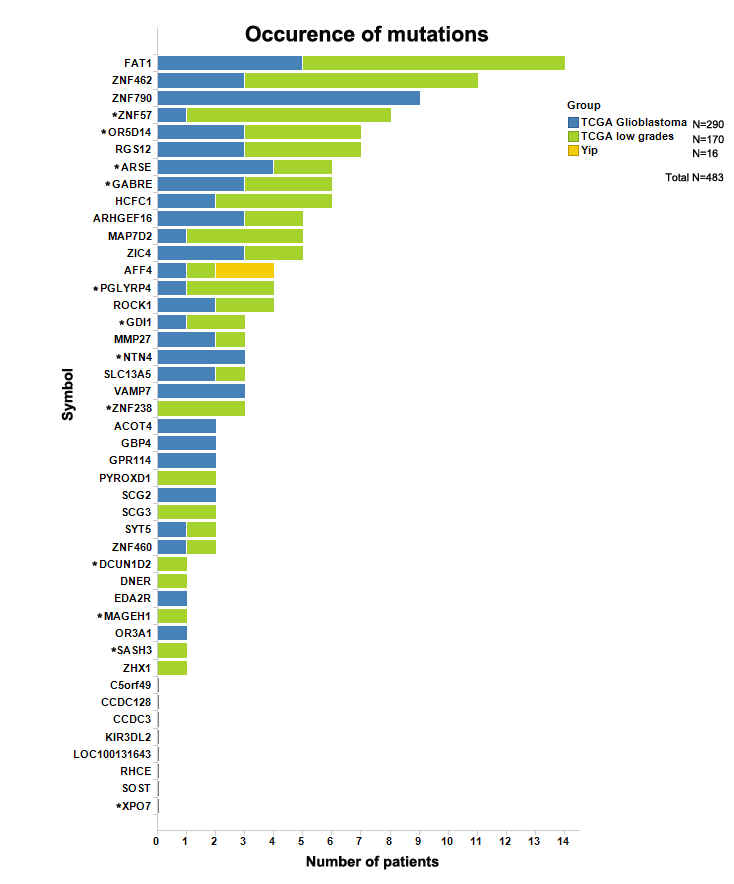

Supplement: Supplementary file 1 — Supplementary material 1 (PNG 38 kb) [file 11060_2015_1741_MOESM1_ESM.png]

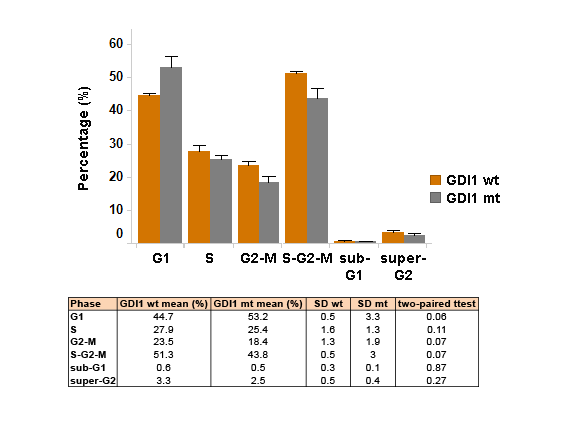

Supplement: Supplementary file 2 — Supplementary material 2 (PNG 31 kb) [file 11060_2015_1741_MOESM2_ESM.png]

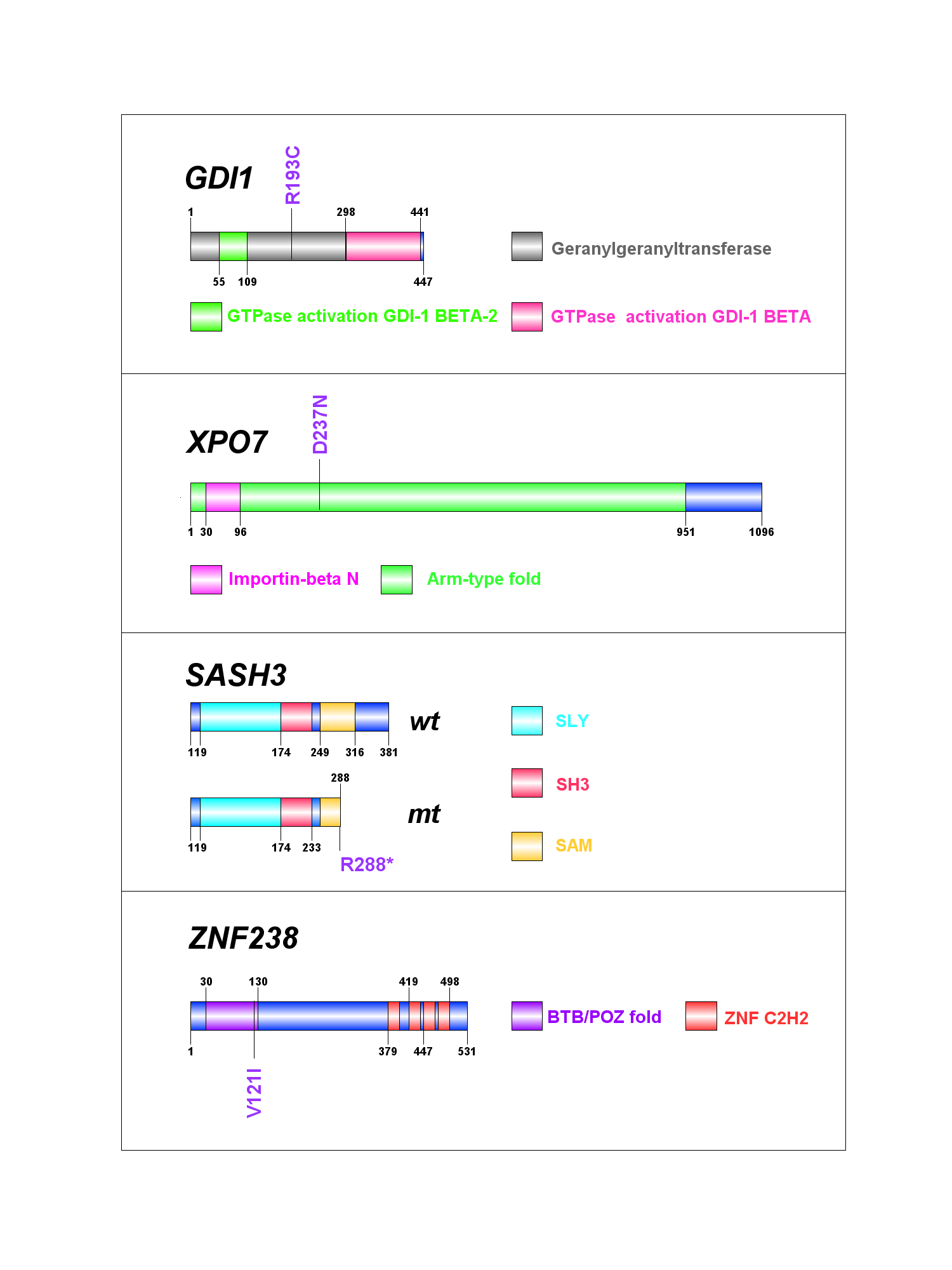

Supplement: Supplementary file 3 — Supplementary material 3 (PNG 86 kb) [file 11060_2015_1741_MOESM3_ESM.png]
