# Supplementary material for: Tumor-specific mutations in low-frequency genes affect their functional properties
Source: J Neurooncol. 2015 Feb 19;122(3):461–70. doi: 10.1007/s11060-015-1741-1 (PMC4436689; doi:10.1007/s11060-015-1741-1)
Supplement: Supplementary file 4 — Supplementary material 4 (DOCX 18 kb) [file 11060_2015_1741_MOESM4_ESM.docx]

**Supplementary Fig.1** Occurrence of identified mutations in external datasets. Of the genes identified by whole genome sequencing, 36 mutations were found in 44 genes in any of the additional 483 tumors of these datasets. Eight mutations were uniquely identified by us. The genes for which functional analyses was performed are marked with a star.

**Supplementary Fig.2**

Cell cycle analysis of cells expressing *GDI1* wildtype or mutant showed that the number of wildtype *GDI1* cells was higher in the S-G2-M phase (7.5%) and lower in the G1 phase (8.5%) compared to mutant *GDI1*. The experiments were performed in triplicate on a BD FACSAria III (BD Biosciences, S. Jose, CA). Histograms are shown for G1, S, G2-M, S-G2-M, sub-G1 (apoptotic) and super-G2 (aneuploid)**.** SD; standard deviation

**Supplementary Fig.3** Protein domain structure of *GDI1*, *XPO7*, *SASH3* and *ZNF238*. (A) *GDI1* consists of three domains: a geranyl-geranyl transferase (residudes 1-298), a GTPase activation GDI1-β2 (residues 55-109) and a GTPase activation GDI1-β domain (residues 298-441). The mutation identified in *GDI1* was located in the geranyl-geranyl transferase domain (c.577C>T, resulting in p.R193C). (B) *XPO7* contains two domains: a N-terminal Importin-beta domain (residues 30-96) and an ARM-type fold domain (residues 1-951). The mutation in *XPO7* was identified in the ARM-type fold domain (c.709G>A resulting in p.D237N). (C) *SASH3* consists of three domains: a SLY motif (residues 19-174), a SH3 motif (residues 174-233) and a SAM motif (249-316). The identified mutation was located in the SAM domain (c.862C>T), partially disrupting the C-terminal region of this domain (p.R288*). (D) *ZNF238* contains a BTB/POZ fold domain (30-130) and four zinc fingers in the C-terminal region, localized to residues 379-401, 419-441, 447-469, 475-498. The mutation was located in the BTB/POZ fold domain (c.361G>A resulting in p.V121I), two amino acids N-terminal from the nuclear localization signal.
