# Supplementary material for: Tumor-specific mutations in low-frequency genes affect their functional properties
Source: J Neurooncol. 2015 Feb 19;122(3):461–70. doi: 10.1007/s11060-015-1741-1 (PMC4436689; doi:10.1007/s11060-015-1741-1)
Supplement: Supplementary file 5 — Supplementary material 5 (DOC 34 kb) [file 11060_2015_1741_MOESM5_ESM.doc]

**Supplementary methods**

**DNA extraction and sequencing**

Whole-genome sequencing was performed by Complete Genomics (Mountain View, US) using 5 µg DNA. Reads were aligned to the reference genome, build 36.1 (HG18). Somatic variations were identified for each patient using the whole-genome data from the matched normal DNA. For this manuscript we focused only variants that result in changes in the primary protein sequence (non-synonymous), were absent in dbSNP version 130 and had a somatic score ≥ -20. The somatic score represents a value of the likelihood that a mutation is somatic (<http://cgatools.sourceforge.net/docs/1.1.0>). A somatic score of 0 is associated with a false discovery rate (FDR) of less than 1%; a somatic score of -20 is associated with a FDR of 4% (see supplementary table 2, (<http://cgatools.sourceforge.net/docs/1.1.0/cgatools-methods.pdf>).

An additional 39 ODs were used for targeted resequencing in which a library was constructed with 10 base pairs upstream and downstream of all exons of mutated genes, including splice sites (supplementary table 3). Single nucleotide variation (SNV) and deletion/insertion polymorphisms (DIP) detection of the targeted resequencing data was done using CLC Bio Genomics Workbench, version 4.9 (Aarhus, Denmark) and filtered using a minimum of 4x coverage and a variant frequency > 35%.

**Cell lines and sorting**

Stably transfected HOG cells were created using geneticin selection (500 μg/ml) for 6 weeks followed by FACS sorting on a BD FACSAria III (BD Biosciences, S. Jose, CA) for GFP-positive cells (FITC channel, 488 nm laser with emission filters LP502 and BP530/30) . Sorting was performed with a 100µm nozzle and a pressure of 20psi.

Protein motifs were determined by SMART, Motif and MutationTaster. Polyphen-2 was used to predict the functional effect of the mutations.

For the cell cycle analysis, cells (300.000/well) were plated in a six-well Greiner plate (Greiner Bio-One, Alphen a/d Rijn, the Netherlands)..Then, cell pellets were collected at 16h after plating, washed with PBS and fixed with 70% ethanol. After one wash with PBS, cells were stained with propidium iodide (PI) solution containing PBS and RNase at room temperature for 30 minutes. The cell cycle experiments were performed in triplicate on a BD FACSAria III (BD Biosciences, S. Jose, CA).
