# Supplementary material for: Tumor-specific mutations in low-frequency genes affect their functional properties
Source: J Neurooncol. 2015 Feb 19;122(3):461–70. doi: 10.1007/s11060-015-1741-1 (PMC4436689; doi:10.1007/s11060-015-1741-1)
Supplement: Supplementary file 6 — Supplementary material 6 (DOC 76 kb) [file 11060_2015_1741_MOESM6_ESM.doc]

Supplementary table 1. Patient characteristics

Abbreviations; m: men, f: female, ODII: oligodendroglioma grade II, ODIII: oligodendroglioma grade III, OA: oligoastrocytoma, LOH: loss of heterozygosity, WGS: whole genome sequencing, TRS: targeted resequencing.
