# Supplementary material for: Tumor-specific mutations in low-frequency genes affect their functional properties
Source: J Neurooncol. 2015 Feb 19;122(3):461–70. doi: 10.1007/s11060-015-1741-1 (PMC4436689; doi:10.1007/s11060-015-1741-1)
Supplement: Supplementary file 7 — Supplementary material 7 (DOC 113 kb) [file 11060_2015_1741_MOESM7_ESM.doc]

Supplementary table 2.Validated somatic mutations from whole-genome sequencing of three ODs grade III

Abbreviations; del: deletion, sub: substitution
