# Supplementary material for: Tumor-specific mutations in low-frequency genes affect their functional properties
Source: J Neurooncol. 2015 Feb 19;122(3):461–70. doi: 10.1007/s11060-015-1741-1 (PMC4436689; doi:10.1007/s11060-015-1741-1)
Supplement: Supplementary file 8 — Supplementary material 8 (DOC 829 kb) [file 11060_2015_1741_MOESM8_ESM.doc]

Supplementary table 3. Capture design for targeted resequencing

| **hg19** | **Gene** |
| --- | --- |
| chrX:65858827 -65858893 | EDA2R1 |
| chrX:65835766 -65835883 | EDA2R1 |
| chrX:65824880 -65825078 | EDA2R1 |
| chrX:65824253 -65824358 | EDA2R1 |
| chrX:65822465 -65822649 | EDA2R1 |
| chrX:65819871 -65819947 | EDA2R1 |
| chrX:65819316 -65819718 | EDA2R1 |
| chrX:65819306 -65819712 | EDA2R1 |
| chrX:65815469 -65817945 | EDA2R1 |
| chrX:55478528 -55480008 | MAGEH1 |
| chrX:2882255 -2882321 | ARSE |
| chrX:2878409 -2878519 | ARSE |
| chrX:2877634 -2877939 | ARSE |
| chrX:2876305 -2876486 | ARSE |
| chrX:2873447 -2873588 | ARSE |
| chrX:2871174 -2871316 | ARSE |
| chrX:2870862 -2871043 | ARSE |
| chrX:2867335 -2867778 | ARSE |
| chrX:2864029 -2864185 | ARSE |
| chrX:2861096 -2861250 | ARSE |
| chrX:2856126 -2856308 | ARSE |
| chrX:2854773 -2854914 | ARSE |
| chrX:2852847 -2853241 | ARSE |
| chrX:20134858 -20135026 | MAP7D2 |
| chrX:20082836 -20082933 | MAP7D2 |
| chrX:20081522 -20081705 | MAP7D2 |
| chrX:20074788 -20074919 | MAP7D2 |
| chrX:20073842 -20073960 | MAP7D2 |
| chrX:20070982 -20071116 | MAP7D2 |
| chrX:20070772 -20070795 | MAP7D2 |
| chrX:20070260 -20070281 | MAP7D2 |
| chrX:20068932 -20069074 | MAP7D2 |
| chrX:20062912 -20062933 | MAP7D2 |
| chrX:20062472 -20062652 | MAP7D2 |
| chrX:20060610 -20060757 | MAP7D2 |
| chrX:20043789 -20044080 | MAP7D2 |
| chrX:20043059 -20043211 | MAP7D2 |
| chrX:20039456 -20039677 | MAP7D2 |
| chrX:20034202 -20034453 | MAP7D2 |
| chrX:20033331 -20033455 | MAP7D2 |
| chrX:20031677 -20031753 | MAP7D2 |
| chrX:20031162 -20031260 | MAP7D2 |
| chrX:20030457 -20030663 | MAP7D2 |
| chrX:20028885 -20029180 | MAP7D2 |
| chrX:20024821 -20026526 | MAP7D2 |
| chrX:154824731-154826637 | VAMP7 HG18 |
| chrX:154822549-154822661 | VAMP7 HG18 |
| chrX:154802661-154802748 | VAMP7 HG18 |
| chrX:154783345-154783455 | VAMP7 HG18 |
| chrX:154780960-154781117 | VAMP7 HG18 |
| chrX:154778470-154778547 | VAMP7 HG18 |
| chrX:154772305-154772500 | VAMP7 HG18 |
| chrX:154764140-154764315 | VAMP7 HG18 |
| chrX:153670857 -153671824 | GDI1 |
| chrX:153670711 -153671075 | GDI1 |
| chrX:153670448 -153671822 | GDI1 |
| chrX:153670103 -153670612 | GDI1 |
| chrX:153669960 -153670455 | GDI1 |
| chrX:153669433 -153669747 | GDI1 |
| chrX:153668988 -153669552 | GDI1 |
| chrX:153668330 -153668863 | GDI1 |
| chrX:153668278 -153670151 | GDI1 |
| chrX:153668047 -153668154 | GDI1 |
| chrX:153667342 -153667607 | GDI1 |
| chrX:153667101 -153667220 | GDI1 |
| chrX:153666552 -153666742 | GDI1 |
| chrX:153666342 -153666986 | GDI1 |
| chrX:153665996 -153666298 | GDI1 |
| chrX:153665256 -153665655 | GDI1 |
| chrX:153236089 -153237268 | HCFC1 |
| chrX:153230019 -153230187 | HCFC1 |
| chrX:153229565 -153229745 | HCFC1 |
| chrX:153228666 -153228894 | HCFC1 |
| chrX:153227663 -153227767 | HCFC1 |
| chrX:153226983 -153227109 | HCFC1 |
| chrX:153225676 -153225875 | HCFC1 |
| chrX:153225243 -153225622 | HCFC1 |
| chrX:153224772 -153224952 | HCFC1 |
| chrX:153224010 -153224227 | HCFC1 |
| chrX:153223466 -153223710 | HCFC1 |
| chrX:153223223 -153223347 | HCFC1 |
| chrX:153222755 -153222994 | HCFC1 |
| chrX:153222359 -153222521 | HCFC1 |
| chrX:153222066 -153222224 | HCFC1 |
| chrX:153221632 -153221872 | HCFC1 |
| chrX:153219507 -153221003 | HCFC1 |
| chrX:153219048 -153219231 | HCFC1 |
| chrX:153217955 -153218551 | HCFC1 |
| chrX:153217282 -153217622 | HCFC1 |
| chrX:153217030 -153217168 | HCFC1 |
| chrX:153216791 -153216948 | HCFC1 |
| chrX:153216254 -153216459 | HCFC1 |
| chrX:153215684 -153216004 | HCFC1 |
| chrX:153214994 -153215077 | HCFC1 |
| chrX:153212994 -153214847 | HCFC1 |
| chrX:151143032 -151143162 | GABRE |
| chrX:151139602 -151141142 | GABRE |
| chrX:151138647 -151139034 | GABRE |
| chrX:151138314 -151138884 | GABRE |
| chrX:151137861 -151138218 | GABRE |
| chrX:151130885 -151131125 | GABRE |
| chrX:151129687 -151129847 | GABRE |
| chrX:151128301 -151128458 | GABRE |
| chrX:151127453 -151127510 | GABRE |
| chrX:151127108 -151127631 | GABRE |
| chrX:151125745 -151125968 | GABRE |
| chrX:151125649 -151125994 | GABRE |
| chrX:151124499 -151124952 | GABRE |
| chrX:151124170 -151128205 | GABRE |
| chrX:151123566 -151124049 | GABRE |
| chrX:151121587 -151123701 | GABRE |
| chrX:151121586 -151123566 | GABRE |
| chrX:128927608 -128929187 | SASH3 |
| chrX:128926955 -128927125 | SASH3 |
| chrX:128926298 -128926822 | SASH3 |
| chrX:128926294 -128926462 | SASH3 |
| chrX:128924906 -128925071 | SASH3 |
| chrX:128922397 -128922563 | SASH3 |
| chrX:128921941 -128922136 | SASH3 |
| chrX:128913945 -128914140 | SASH3 |
| chr9:22008787 -22009290 | CDKN2B |
| chr9:22008664 -22009281 | CDKN2B |
| chr9:22005892 -22006256 | CDKN2B |
| chr9:21994810 -21995310 | CDKN2A |
| chr9:21994128 -21994500 | CDKN2A |
| chr9:21993871 -21994077 | CDKN2A |
| chr9:21989326 -21989964 | CDKN2A |
| chr9:21974667 -21975107 | CDKN2A |
| chr9:21974393 -21974836 | CDKN2A |
| chr9:21971318 -21971409 | CDKN2A |
| chr9:21970891 -21971341 | CDKN2A |
| chr9:21970705 -21971217 | CDKN2A |
| chr9:21970431 -21970512 | CDKN2A |
| chr9:21969558 -21969802 | CDKN2A |
| chr9:21968564 -21968780 | CDKN2A |
| chr9:21967742 -21968251 | CDKN2A |
| chr9:139440168 -139440324 | NOTCH1 |
| chr9:139438466 -139438564 | NOTCH1 |
| chr9:139418159 -139418441 | NOTCH1 |
| chr9:139417070 -139417650 | NOTCH1 |
| chr9:139413885 -139414027 | NOTCH1 |
| chr9:139413033 -139413286 | NOTCH1 |
| chr9:139412579 -139412754 | NOTCH1 |
| chr9:139412194 -139412399 | NOTCH1 |
| chr9:139411714 -139411847 | NOTCH1 |
| chr9:139410423 -139410556 | NOTCH1 |
| chr9:139409925 -139410178 | NOTCH1 |
| chr9:139409732 -139409862 | NOTCH1 |
| chr9:139408952 -139409164 | NOTCH1 |
| chr9:139407834 -139407999 | NOTCH1 |
| chr9:139407463 -139407596 | NOTCH1 |
| chr9:139405594 -139405733 | NOTCH1 |
| chr9:139405095 -139405267 | NOTCH1 |
| chr9:139404175 -139404423 | NOTCH1 |
| chr9:139403312 -139403533 | NOTCH1 |
| chr9:139402674 -139402847 | NOTCH1 |
| chr9:139402397 -139402601 | NOTCH1 |
| chr9:139401747 -139401899 | NOTCH1 |
| chr9:139401158 -139401435 | NOTCH1 |
| chr9:139400969 -139401101 | NOTCH1 |
| chr9:139399752 -139400343 | NOTCH1 |
| chr9:139399115 -139399566 | NOTCH1 |
| chr9:139397624 -139397792 | NOTCH1 |
| chr9:139396888 -139397237 | NOTCH1 |
| chr9:139396714 -139396950 | NOTCH1 |
| chr9:139396443 -139396550 | NOTCH1 |
| chr9:139396190 -139396375 | NOTCH1 |
| chr9:139394994 -139395309 | NOTCH1 |
| chr9:139393554 -139393721 | NOTCH1 |
| chr9:139393341 -139393458 | NOTCH1 |
| chr9:139389886 -139392020 | NOTCH1 |
| chr9:109773094 -109775925 | ZNF462 |
| chr9:109771460 -109771959 | ZNF462 |
| chr9:109765565 -109765717 | ZNF462 |
| chr9:109757791 -109757973 | ZNF462 |
| chr9:109746457 -109746700 | ZNF462 |
| chr9:109736408 -109737261 | ZNF462 |
| chr9:109734276 -109734563 | ZNF462 |
| chr9:109701187 -109701398 | ZNF462 |
| chr9:109697774 -109697912 | ZNF462 |
| chr9:109695446 -109695520 | ZNF462 |
| chr9:109694879 -109695096 | ZNF462 |
| chr9:109694717 -109694753 | ZNF462 |
| chr9:109694534 -109694840 | ZNF462 |
| chr9:109693116 -109693328 | ZNF462 |
| chr9:109692796 -109692980 | ZNF462 |
| chr9:109689196 -109689455 | ZNF462 |
| chr9:109686404 -109692050 | ZNF462 |
| chr9:109685625 -109685894 | ZNF462 |
| chr9:109625368 -109625646 | ZNF462 |
| chr8:21862496 -21864106 | XPO7 |
| chr8:21861404 -21861551 | XPO7 |
| chr8:21860486 -21860838 | XPO7 |
| chr8:21859613 -21859793 | XPO7 |
| chr8:21857037 -21857195 | XPO7 |
| chr8:21856592 -21856826 | XPO7 |
| chr8:21856256 -21856358 | XPO7 |
| chr8:21852993 -21853120 | XPO7 |
| chr8:21851864 -21851972 | XPO7 |
| chr8:21849227 -21849495 | XPO7 |
| chr8:21848312 -21848440 | XPO7 |
| chr8:21847845 -21848132 | XPO7 |
| chr8:21845849 -21846604 | XPO7 |
| chr8:21845285 -21845378 | XPO7 |
| chr8:21844642 -21844797 | XPO7 |
| chr8:21843087 -21843212 | XPO7 |
| chr8:21842147 -21842360 | XPO7 |
| chr8:21840141 -21840333 | XPO7 |
| chr8:21839232 -21839398 | XPO7 |
| chr8:21837585 -21837724 | XPO7 |
| chr8:21835271 -21835364 | XPO7 |
| chr8:21833857 -21834042 | XPO7 |
| chr8:21832171 -21832295 | XPO7 |
| chr8:21829377 -21829489 | XPO7 |
| chr8:21827645 -21827831 | XPO7 |
| chr8:21826984 -21827417 | XPO7 |
| chr8:21824358 -21824524 | XPO7 |
| chr8:21823739 -21823834 | XPO7 |
| chr8:21821650 -21821846 | XPO7 |
| chr8:21778915 -21779147 | XPO7 |
| chr8:21777170 -21777309 | XPO7 |
| chr8:128752632 -128753684 | MYC |
| chr8:128750484 -128751275 | MYC |
| chr8:128748670 -128748879 | MYC |
| chr8:124287570 -124287791 | ZHX1 |
| chr8:124286447 -124286745 | ZHX1 |
| chr8:124286352 -124286734 | ZHX1 |
| chr8:124285980 -124286247 | ZHX1 |
| chr8:124279503 -124279641 | ZHX1 |
| chr8:124269263 -124269467 | ZHX1 |
| chr8:124265552 -124268421 | ZHX1 |
| chr8:124260687 -124262570 | ZHX1 |
| chr7:55323937 -55324323 | EGFR |
| chr7:55272939 -55274331 | EGFR |
| chr7:55270200 -55270779 | EGFR |
| chr7:55269418 -55269485 | EGFR |
| chr7:55268871 -55269058 | EGFR |
| chr7:55267999 -55268505 | EGFR |
| chr7:55266400 -55266566 | EGFR |
| chr7:55260449 -55260544 | EGFR |
| chr7:55259402 -55259577 | EGFR |
| chr7:55248976 -55249181 | EGFR |
| chr7:55242405 -55242523 | EGFR |
| chr7:55241604 -55241746 | EGFR |
| chr7:55240666 -55240827 | EGFR |
| chr7:55240529 -55240631 | EGFR |
| chr7:55238858 -55238916 | EGFR |
| chr7:55237990 -55238748 | EGFR |
| chr7:55236206 -55236338 | EGFR |
| chr7:55232963 -55233140 | EGFR |
| chr7:55231416 -55231526 | EGFR |
| chr7:55229182 -55229334 | EGFR |
| chr7:55227822 -55228041 | EGFR |
| chr7:55225346 -55225456 | EGFR |
| chr7:55224442 -55224654 | EGFR |
| chr7:55224216 -55224362 | EGFR |
| chr7:55223513 -55223649 | EGFR |
| chr7:55221694 -55221855 | EGFR |
| chr7:55220229 -55220367 | EGFR |
| chr7:55218977 -55219065 | EGFR |
| chr7:55214289 -55214443 | EGFR |
| chr7:55210988 -55211191 | EGFR |
| chr7:55209969 -55210140 | EGFR |
| chr7:55186739 -55187104 | EGFR |
| chr7:55177406 -55177661 | EGFR |
| chr7:55111516 -55111752 | EGFR |
| chr7:55086704 -55087068 | EGFR |
| chr7:140624356 -140624574 | BRAF |
| chr7:140549901 -140550022 | BRAF |
| chr7:140533851 -140534682 | BRAF |
| chr7:140508682 -140508805 | BRAF |
| chr7:140508027 -140508126 | BRAF |
| chr7:140507750 -140507872 | BRAF |
| chr7:140501202 -140501370 | BRAF |
| chr7:140500152 -140500291 | BRAF |
| chr7:140494098 -140494277 | BRAF |
| chr7:140487338 -140487394 | BRAF |
| chr7:140482811 -140482967 | BRAF |
| chr7:140481366 -140481503 | BRAF |
| chr7:140477781 -140477885 | BRAF |
| chr7:140476702 -140476898 | BRAF |
| chr7:140453977 -140454043 | BRAF |
| chr7:140453065 -140453203 | BRAF |
| chr7:140449077 -140449228 | BRAF |
| chr7:140447156 -140447257 | BRAF |
| chr7:140439602 -140439756 | BRAF |
| chr7:140434269 -140434580 | BRAF |
| chr6:32577994 -32578063 | HLADR-B5 |
| chr6:32576093 -32576263 | HLADR-B5 |
| chr6:32557410 -32557635 | HLADR-B5 |
| chr6:32551876 -32552165 | HLADR-B5 |
| chr6:32549324 -32549625 | HLADR-B5 |
| chr6:32548513 -32548643 | HLADR-B5 |
| chr6:32548014 -32548057 | HLADR-B5 |
| chr6:32546536 -32546891 | HLADR-B5 |
| chr6:2903425 -2903524 | SERPINB9 |
| chr6:2900668 -2900865 | SERPINB9 |
| chr6:2896277 -2896434 | SERPINB9 |
| chr6:2895615 -2895752 | SERPINB9 |
| chr6:2893635 -2893797 | SERPINB9 |
| chr6:2892057 -2892232 | SERPINB9 |
| chr6:2887490 -2890814 | SERPINB9 |
| chr5:7850978 -7851613 | C5ORF49 |
| chr5:7835485 -7835621 | C5ORF49 |
| chr5:7831501 -7832146 | C5ORF49 |
| chr5:7830481 -7832152 | C5ORF49 |
| chr5:67593230 -67593659 | PIK3R1 |
| chr5:67591965 -67592179 | PIK3R1 |
| chr5:67591238 -67591581 | PIK3R1 |
| chr5:67590966 -67591162 | PIK3R1 |
| chr5:67590354 -67590516 | PIK3R1 |
| chr5:67589527 -67589672 | PIK3R1 |
| chr5:67589121 -67589321 | PIK3R1 |
| chr5:67588919 -67589230 | PIK3R1 |
| chr5:67588501 -67589037 | PIK3R1 |
| chr5:67588386 -67588567 | PIK3R1 |
| chr5:67588304 -67588361 | PIK3R1 |
| chr5:67586618 -67588199 | PIK3R1 |
| chr5:67586455 -67586672 | PIK3R1 |
| chr5:67584186 -67584589 | PIK3R1 |
| chr5:67576745 -67576844 | PIK3R1 |
| chr5:67576125 -67576567 | PIK3R1 |
| chr5:67576058 -67576101 | PIK3R1 |
| chr5:67575420 -67575571 | PIK3R1 |
| chr5:67569757 -67569851 | PIK3R1 |
| chr5:67569208 -67569519 | PIK3R1 |
| chr5:67535716 -67535775 | PIK3R1 |
| chr5:67522108 -67522847 | PIK3R1 |
| chr5:67513284 -67513636 | PIK3R1 |
| chr5:67511594 -67511791 | PIK3R1 |
| chr5:67511538 -67511787 | PIK3R1 |
| chr5:132298941 -132299336 | AFF4 |
| chr5:132273331 -132273442 | AFF4 |
| chr5:132272749 -132272895 | AFF4 |
| chr5:132269829 -132270643 | AFF4 |
| chr5:132267860 -132267924 | AFF4 |
| chr5:132262803 -132263264 | AFF4 |
| chr5:132262383 -132262909 | AFF4 |
| chr5:132240050 -132240106 | AFF4 |
| chr5:132239543 -132239882 | AFF4 |
| chr5:132238124 -132238189 | AFF4 |
| chr5:132236554 -132236784 | AFF4 |
| chr5:132235269 -132235343 | AFF4 |
| chr5:132234786 -132234843 | AFF4 |
| chr5:132233912 -132234094 | AFF4 |
| chr5:132232005 -132232942 | AFF4 |
| chr5:132228712 -132228820 | AFF4 |
| chr5:132227499 -132228106 | AFF4 |
| chr5:132224761 -132224875 | AFF4 |
| chr5:132223779 -132223862 | AFF4 |
| chr5:132223528 -132223684 | AFF4 |
| chr5:132223203 -132223294 | AFF4 |
| chr5:132221992 -132222105 | AFF4 |
| chr5:132220758 -132220821 | AFF4 |
| chr5:132219022 -132219262 | AFF4 |
| chr5:132211061 -132216888 | AFF4 |
| chr4:55161282 -55161624 | PDGFRA |
| chr4:55156470 -55156731 | PDGFRA |
| chr4:55155166 -55155291 | PDGFRA |
| chr4:55154956 -55155075 | PDGFRA |
| chr4:55153587 -55153718 | PDGFRA |
| chr4:55151998 -55152140 | PDGFRA |
| chr4:55151528 -55151663 | PDGFRA |
| chr4:55147738 -55148155 | PDGFRA |
| chr4:55146473 -55146935 | PDGFRA |
| chr4:55144976 -55145148 | PDGFRA |
| chr4:55144519 -55144692 | PDGFRA |
| chr4:55144053 -55144423 | PDGFRA |
| chr4:55143333 -55143669 | PDGFRA |
| chr4:55142908 -55142951 | PDGFRA |
| chr4:55140998 -55141150 | PDGFRA |
| chr4:55140688 -55140802 | PDGFRA |
| chr4:55139694 -55139907 | PDGFRA |
| chr4:55138551 -55138697 | PDGFRA |
| chr4:55136790 -55136925 | PDGFRA |
| chr4:55133709 -55133918 | PDGFRA |
| chr4:55133446 -55133637 | PDGFRA |
| chr4:55129824 -55131530 | PDGFRA |
| chr4:55127252 -55127589 | PDGFRA |
| chr4:55124914 -55124994 | PDGFRA |
| chr4:55109732 -55109849 | PDGFRA |
| chr4:55106195 -55106292 | PDGFRA |
| chr4:55096479 -55096783 | PDGFRA |
| chr4:55096000 -55096116 | PDGFRA |
| chr4:55095254 -55095592 | PDGFRA |
| chr4:3441172 -3441650 | RGS12 |
| chr4:3432124 -3433987 | RGS12 |
| chr4:3430275 -3430448 | RGS12 |
| chr4:3429807 -3430335 | RGS12 |
| chr4:3429806 -3429906 | RGS12 |
| chr4:3428323 -3428491 | RGS12 |
| chr4:3427163 -3427297 | RGS12 |
| chr4:3425232 -3425602 | RGS12 |
| chr4:3424622 -3424715 | RGS12 |
| chr4:3424093 -3424307 | RGS12 |
| chr4:3422105 -3422455 | RGS12 |
| chr4:3419105 -3420004 | RGS12 |
| chr4:3419087 -3421247 | RGS12 |
| chr4:3418630 -3418829 | RGS12 |
| chr4:3417695 -3417858 | RGS12 |
| chr4:3415789 -3416581 | RGS12 |
| chr4:3415623 -3415655 | RGS12 |
| chr4:3414946 -3415440 | RGS12 |
| chr4:3414703 -3415978 | RGS12 |
| chr4:3387688 -3388174 | RGS12 |
| chr4:3387416 -3387530 | RGS12 |
| chr4:3387398 -3387553 | RGS12 |
| chr4:3387321 -3387388 | RGS12 |
| chr4:3386863 -3387161 | RGS12 |
| chr4:3379887 -3380328 | RGS12 |
| chr4:3371596 -3372058 | RGS12 |
| chr4:3344654 -3344926 | RGS12 |
| chr4:3344132 -3344315 | RGS12 |
| chr4:3317785 -3319788 | RGS12 |
| chr4:3315864 -3316686 | RGS12 |
| chr4:3294745 -3294836 | RGS12 |
| chr4:187647498 -187647886 | FAT1 |
| chr4:187644808 -187645019 | FAT1 |
| chr4:187627707 -187631009 | FAT1 |
| chr4:187584443 -187584777 | FAT1 |
| chr4:187560866 -187560947 | FAT1 |
| chr4:187557729 -187558078 | FAT1 |
| chr4:187557169 -187557399 | FAT1 |
| chr4:187554828 -187554987 | FAT1 |
| chr4:187549632 -187549927 | FAT1 |
| chr4:187549289 -187549528 | FAT1 |
| chr4:187538852 -187542939 | FAT1 |
| chr4:187538149 -187538365 | FAT1 |
| chr4:187535335 -187535508 | FAT1 |
| chr4:187534253 -187534506 | FAT1 |
| chr4:187532530 -187532939 | FAT1 |
| chr4:187530945 -187531234 | FAT1 |
| chr4:187530327 -187530484 | FAT1 |
| chr4:187527214 -187527377 | FAT1 |
| chr4:187525521 -187525738 | FAT1 |
| chr4:187524320 -187525141 | FAT1 |
| chr4:187524047 -187524198 | FAT1 |
| chr4:187522413 -187522864 | FAT1 |
| chr4:187521042 -187521524 | FAT1 |
| chr4:187519671 -187519985 | FAT1 |
| chr4:187519116 -187519291 | FAT1 |
| chr4:187518826 -187518956 | FAT1 |
| chr4:187518255 -187518435 | FAT1 |
| chr4:187517684 -187518395 | FAT1 |
| chr4:187516833 -187516990 | FAT1 |
| chr4:187513837 -187513916 | FAT1 |
| chr4:187511649 -187511859 | FAT1 |
| chr4:187511512 -187511567 | FAT1 |
| chr4:187508927 -187510384 | FAT1 |
| chr4:15602850 -15603190 | CC2D2A |
| chr4:15601142 -15601339 | CC2D2A |
| chr4:15599020 -15599098 | CC2D2A |
| chr4:15597485 -15597840 | CC2D2A |
| chr4:15591158 -15591312 | CC2D2A |
| chr4:15589429 -15589562 | CC2D2A |
| chr4:15587770 -15587879 | CC2D2A |
| chr4:15581581 -15581804 | CC2D2A |
| chr4:15575763 -15575959 | CC2D2A |
| chr4:15572011 -15572129 | CC2D2A |
| chr4:15570881 -15571022 | CC2D2A |
| chr4:15569290 -15569419 | CC2D2A |
| chr4:15568990 -15569115 | CC2D2A |
| chr4:15564968 -15565155 | CC2D2A |
| chr4:15562144 -15562255 | CC2D2A |
| chr4:15560778 -15560890 | CC2D2A |
| chr4:15558917 -15559140 | CC2D2A |
| chr4:15556685 -15556843 | CC2D2A |
| chr4:15554771 -15554938 | CC2D2A |
| chr4:15552351 -15552613 | CC2D2A |
| chr4:15545192 -15545594 | CC2D2A |
| chr4:15542450 -15542647 | CC2D2A |
| chr4:15539512 -15539770 | CC2D2A |
| chr4:15538533 -15538709 | CC2D2A |
| chr4:15534806 -15535086 | CC2D2A |
| chr4:15530233 -15530359 | CC2D2A |
| chr4:15529060 -15529289 | CC2D2A |
| chr4:15518238 -15518389 | CC2D2A |
| chr4:15517481 -15517637 | CC2D2A |
| chr4:15516320 -15516502 | CC2D2A |
| chr4:15512860 -15513308 | CC2D2A |
| chr4:15511752 -15511873 | CC2D2A |
| chr4:15504435 -15504556 | CC2D2A |
| chr4:15504042 -15504150 | CC2D2A |
| chr4:15482811 -15483951 | CC2D2A |
| chr4:15482318 -15482461 | CC2D2A |
| chr4:15480837 -15480962 | CC2D2A |
| chr4:15480337 -15480440 | CC2D2A |
| chr4:15477516 -15477605 | CC2D2A |
| chr4:15474836 -15474889 | CC2D2A |
| chr4:15471505 -15471691 | CC2D2A |
| chr4:15471479 -15471727 | CC2D2A |
| chr4:123283176 -123283917 | KIAA1109 |
| chr4:123280702 -123280887 | KIAA1109 |
| chr4:123277766 -123277920 | KIAA1109 |
| chr4:123276941 -123277155 | KIAA1109 |
| chr4:123274959 -123275182 | KIAA1109 |
| chr4:123274049 -123274320 | KIAA1109 |
| chr4:123271002 -123271239 | KIAA1109 |
| chr4:123270595 -123270807 | KIAA1109 |
| chr4:123270058 -123270480 | KIAA1109 |
| chr4:123269712 -123269882 | KIAA1109 |
| chr4:123268667 -123268944 | KIAA1109 |
| chr4:123267768 -123267925 | KIAA1109 |
| chr4:123265527 -123265776 | KIAA1109 |
| chr4:123264555 -123264775 | KIAA1109 |
| chr4:123260352 -123260573 | KIAA1109 |
| chr4:123258026 -123258312 | KIAA1109 |
| chr4:123257238 -123257518 | KIAA1109 |
| chr4:123255489 -123255710 | KIAA1109 |
| chr4:123254774 -123254974 | KIAA1109 |
| chr4:123252471 -123252706 | KIAA1109 |
| chr4:123249017 -123249522 | KIAA1109 |
| chr4:123247000 -123247082 | KIAA1109 |
| chr4:123246830 -123246949 | KIAA1109 |
| chr4:123246375 -123246485 | KIAA1109 |
| chr4:123245572 -123245701 | KIAA1109 |
| chr4:123239292 -123239435 | KIAA1109 |
| chr4:123237877 -123238027 | KIAA1109 |
| chr4:123237493 -123237713 | KIAA1109 |
| chr4:123236607 -123236853 | KIAA1109 |
| chr4:123234773 -123234852 | KIAA1109 |
| chr4:123230411 -123230629 | KIAA1109 |
| chr4:123229094 -123229325 | KIAA1109 |
| chr4:123227022 -123227210 | KIAA1109 |
| chr4:123225943 -123226148 | KIAA1109 |
| chr4:123222445 -123222562 | KIAA1109 |
| chr4:123210232 -123210357 | KIAA1109 |
| chr4:123207686 -123207950 | KIAA1109 |
| chr4:123202681 -123202939 | KIAA1109 |
| chr4:123200920 -123201146 | KIAA1109 |
| chr4:123197117 -123197354 | KIAA1109 |
| chr4:123195488 -123195601 | KIAA1109 |
| chr4:123193305 -123193570 | KIAA1109 |
| chr4:123192163 -123192889 | KIAA1109 |
| chr4:123187928 -123188123 | KIAA1109 |
| chr4:123185397 -123185592 | KIAA1109 |
| chr4:123184587 -123184765 | KIAA1109 |
| chr4:123183909 -123184148 | KIAA1109 |
| chr4:123179846 -123180008 | KIAA1109 |
| chr4:123178400 -123178660 | KIAA1109 |
| chr4:123176276 -123176448 | KIAA1109 |
| chr4:123175950 -123176120 | KIAA1109 |
| chr4:123175340 -123175511 | KIAA1109 |
| chr4:123171507 -123171738 | KIAA1109 |
| chr4:123170630 -123170847 | KIAA1109 |
| chr4:123168303 -123168853 | KIAA1109 |
| chr4:123167828 -123167975 | KIAA1109 |
| chr4:123167297 -123167463 | KIAA1109 |
| chr4:123166165 -123166305 | KIAA1109 |
| chr4:123164999 -123165192 | KIAA1109 |
| chr4:123164143 -123164233 | KIAA1109 |
| chr4:123160661 -123161518 | KIAA1109 |
| chr4:123159237 -123159515 | KIAA1109 |
| chr4:123155918 -123156188 | KIAA1109 |
| chr4:123151076 -123151376 | KIAA1109 |
| chr4:123150266 -123150405 | KIAA1109 |
| chr4:123147852 -123148000 | KIAA1109 |
| chr4:123145646 -123145842 | KIAA1109 |
| chr4:123145643 -123148912 | KIAA1109 |
| chr4:123141478 -123141635 | KIAA1109 |
| chr4:123140485 -123140732 | KIAA1109 |
| chr4:123132032 -123132260 | KIAA1109 |
| chr4:123130963 -123131089 | KIAA1109 |
| chr4:123130304 -123130502 | KIAA1109 |
| chr4:123128686 -123128803 | KIAA1109 |
| chr4:123128255 -123128430 | KIAA1109 |
| chr4:123122154 -123122291 | KIAA1109 |
| chr4:123120504 -123120615 | KIAA1109 |
| chr4:123118341 -123118439 | KIAA1109 |
| chr4:123117780 -123117956 | KIAA1109 |
| chr4:123113374 -123113544 | KIAA1109 |
| chr4:123111145 -123111244 | KIAA1109 |
| chr4:123109040 -123109253 | KIAA1109 |
| chr4:123108576 -123108677 | KIAA1109 |
| chr4:123107181 -123107387 | KIAA1109 |
| chr4:123096995 -123097079 | KIAA1109 |
| chr4:123095719 -123095817 | KIAA1109 |
| chr4:123094181 -123094317 | KIAA1109 |
| chr4:123091748 -123091909 | KIAA1109 |
| chr4:123075170 -123075277 |  |
| chr4:123073478 -123073749 |  |
| chr3:178951872 -178952891 | PIK3CA |
| chr3:178948003 -178948174 | PIK3CA |
| chr3:178947782 -178947919 | PIK3CA |
| chr3:178947050 -178947240 | PIK3CA |
| chr3:178943740 -178943838 | PIK3CA |
| chr3:178942478 -178942619 | PIK3CA |
| chr3:178941859 -178941985 | PIK3CA |
| chr3:178938764 -178938955 | PIK3CA |
| chr3:178937727 -178937850 | PIK3CA |
| chr3:178937329 -178937533 | PIK3CA |
| chr3:178936974 -178937075 | PIK3CA |
| chr3:178935988 -178936132 | PIK3CA |
| chr3:178928209 -178928363 | PIK3CA |
| chr3:178927964 -178928136 | PIK3CA |
| chr3:178927373 -178927498 | PIK3CA |
| chr3:178922281 -178922386 | PIK3CA |
| chr3:178921322 -178921587 | PIK3CA |
| chr3:178919068 -178919338 | PIK3CA |
| chr3:178917468 -178917697 | PIK3CA |
| chr3:178916528 -178916977 | PIK3CA |
| chr3:178867305 -178867514 | PIK3CA |
| chr3:178866301 -178866401 | PIK3CA |
| chr3:178865892 -178866017 | PIK3CA |
| chr3:147124140 -147124657 | ZIC4 |
| chr3:147123685 -147123930 | ZIC4 |
| chr3:147123229 -147123646 | ZIC4 |
| chr3:147123150 -147123317 | ZIC4 |
| chr3:147121741 -147122081 | ZIC4 |
| chr3:147121324 -147121595 | ZIC4 |
| chr3:147120505 -147120609 | ZIC4 |
| chr3:147113629 -147114983 | ZIC4 |
| chr3:147111489 -147111684 | ZIC4 |
| chr3:147109900 -147110227 | ZIC4 |
| chr3:147109801 -147110194 | ZIC4 |
| chr3:147109747 -147110188 | ZIC4 |
| chr3:147108708 -147110730 | ZIC4 |
| chr3:147106769 -147107169 | ZIC4 |
| chr3:147103823 -147106656 | ZIC4 |
| chr20:6758882 -6759920 | BMP2 |
| chr20:6750757 -6751129 | BMP2 |
| chr2:48741844 -48742535 | PPP1R21 |
| chr2:48738469 -48738617 | PPP1R21 |
| chr2:48737144 -48737262 | PPP1R21 |
| chr2:48734340 -48734534 | PPP1R21 |
| chr2:48732693 -48732745 | PPP1R21 |
| chr2:48725622 -48725884 | PPP1R21 |
| chr2:48722900 -48723311 | PPP1R21 |
| chr2:48722808 -48723054 | PPP1R21 |
| chr2:48718147 -48718319 | PPP1R21 |
| chr2:48713760 -48713907 | PPP1R21 |
| chr2:48707053 -48707165 | PPP1R21 |
| chr2:48706613 -48706705 | PPP1R21 |
| chr2:48701812 -48701968 | PPP1R21 |
| chr2:48698440 -48698516 | PPP1R21 |
| chr2:48698408 -48698599 | PPP1R21 |
| chr2:48698216 -48698337 | PPP1R21 |
| chr2:48692622 -48692791 | PPP1R21 |
| chr2:48692066 -48692138 | PPP1R21 |
| chr2:48689236 -48689269 | PPP1R21 |
| chr2:48688267 -48688381 | PPP1R21 |
| chr2:48687224 -48687302 | PPP1R21 |
| chr2:48686883 -48687067 | PPP1R21 |
| chr2:48685131 -48685376 | PPP1R21 |
| chr2:48681685 -48681890 | PPP1R21 |
| chr2:48678137 -48678225 | PPP1R21 |
| chr2:48668258 -48668619 | PPP1R21 |
| chr2:48667898 -48668159 | PPP1R21 |
| chr2:48667727 -48667847 | PPP1R21 |
| chr2:230578854 -230579284 | DNER |
| chr2:230456286 -230456614 | DNER |
| chr2:230453100 -230453214 | DNER |
| chr2:230451400 -230451903 | DNER |
| chr2:230450564 -230450750 | DNER |
| chr2:230444860 -230445070 | DNER |
| chr2:230411653 -230411818 | DNER |
| chr2:230377489 -230377662 | DNER |
| chr2:230341846 -230341979 | DNER |
| chr2:230312022 -230312266 | DNER |
| chr2:230282814 -230282956 | DNER |
| chr2:230271938 -230272071 | DNER |
| chr2:230252971 -230253122 | DNER |
| chr2:230231579 -230231845 | DNER |
| chr2:230222335 -230223377 | DNER |
| chr2:228028713 -228029276 | COL4A4 |
| chr2:228012119 -228012310 | COL4A4 |
| chr2:228009222 -228009284 | COL4A4 |
| chr2:228004867 -228004964 | COL4A4 |
| chr2:227985720 -227985874 | COL4A4 |
| chr2:227984601 -227984665 | COL4A4 |
| chr2:227983351 -227983487 | COL4A4 |
| chr2:227979334 -227979422 | COL4A4 |
| chr2:227976384 -227976439 | COL4A4 |
| chr2:227973930 -227974012 | COL4A4 |
| chr2:227973539 -227973594 | COL4A4 |
| chr2:227973287 -227973348 | COL4A4 |
| chr2:227968678 -227968778 | COL4A4 |
| chr2:227967850 -227967923 | COL4A4 |
| chr2:227967495 -227967574 | COL4A4 |
| chr2:227966571 -227966635 | COL4A4 |
| chr2:227966210 -227966283 | COL4A4 |
| chr2:227964326 -227964415 | COL4A4 |
| chr2:227963400 -227963524 | COL4A4 |
| chr2:227958831 -227959015 | COL4A4 |
| chr2:227954574 -227954683 | COL4A4 |
| chr2:227953359 -227953542 | COL4A4 |
| chr2:227946821 -227946913 | COL4A4 |
| chr2:227945149 -227945275 | COL4A4 |
| chr2:227942600 -227942803 | COL4A4 |
| chr2:227927236 -227927324 | COL4A4 |
| chr2:227924842 -227924969 | COL4A4 |
| chr2:227924111 -227924349 | COL4A4 |
| chr2:227922145 -227922326 | COL4A4 |
| chr2:227920651 -227920841 | COL4A4 |
| chr2:227919300 -227919463 | COL4A4 |
| chr2:227917011 -227917138 | COL4A4 |
| chr2:227915683 -227915884 | COL4A4 |
| chr2:227914774 -227914857 | COL4A4 |
| chr2:227912181 -227912275 | COL4A4 |
| chr2:227907783 -227907910 | COL4A4 |
| chr2:227906854 -227906981 | COL4A4 |
| chr2:227898116 -227898207 | COL4A4 |
| chr2:227896854 -227897002 | COL4A4 |
| chr2:227896651 -227896781 | COL4A4 |
| chr2:227895149 -227895324 | COL4A4 |
| chr2:227892608 -227892735 | COL4A4 |
| chr2:227890508 -227890536 | COL4A4 |
| chr2:227886754 -227886908 | COL4A4 |
| chr2:227876887 -227877023 | COL4A4 |
| chr2:227875019 -227875227 | COL4A4 |
| chr2:227872724 -227873030 | COL4A4 |
| chr2:227867417 -227872314 | COL4A4 |
| chr2:224466993 -224467231 | SCG2 |
| chr2:224466850 -224467012 | SCG2 |
| chr2:224463674 -224464010 | SCG2 |
| chr2:224462137 -224463273 | SCG2 |
| chr2:224461648 -224464024 | SCG2 |
| chr2:209130684 -209130808 | IDH1 |
| chr2:209120243 -209120532 | IDH1 |
| chr2:209119697 -209119822 | IDH1 |
| chr2:209119653 -209119867 | IDH1 |
| chr2:209119501 -209119695 | IDH1 |
| chr2:209119057 -209119192 | IDH1 |
| chr2:209118864 -209119046 | IDH1 |
| chr2:209118745 -209119056 | IDH1 |
| chr2:209118600 -209119073 | IDH1 |
| chr2:209115975 -209116301 | IDH1 |
| chr2:209113083 -209113151 | IDH1 |
| chr2:209112096 -209113394 | IDH1 |
| chr2:209110033 -209110158 | IDH1 |
| chr2:209108141 -209108338 | IDH1 |
| chr2:209106708 -209106879 | IDH1 |
| chr2:209104577 -209105049 | IDH1 |
| chr2:209103785 -209103967 | IDH1 |
| chr2:209100941 -209101903 | IDH1 |
| chr19:9048761 -9049561 | MUC16 |
| chr19:7562823 -7563623 | C19ORF45 |
| chr19:6266581 -6267381 | MLLT1 |
| chr19:62214048 -62214848 |  |
| chr19:61886734 -61887534 |  |
| chr19:60002999 -60003799 |  |
| chr19:59941948 -59942748 |  |
| chr19:59215656 -59216456 |  |
| chr19:58558794 -58559594 | ZSCAN1 |
| chr19:57802057 -57805446 | ZNF460 |
| chr19:57795909 -57796055 | ZNF460 |
| chr19:57791843 -57792214 | ZNF460 |
| chr19:56155911 -56156711 | ZNF581 |
| chr19:55776554 -55777354 | HSPBP1 |
| chr19:55691475 -55691730 | SYT5 |
| chr19:55690321 -55690464 | SYT5 |
| chr19:55689554 -55689746 | SYT5 |
| chr19:55687363 -55687502 | SYT5 |
| chr19:55687067 -55687254 | SYT5 |
| chr19:55686530 -55686717 | SYT5 |
| chr19:55686240 -55686377 | SYT5 |
| chr19:55685875 -55686028 | SYT5 |
| chr19:55684459 -55685062 | SYT5 |
| chr19:55377967 -55378672 | KIR2DS4 |
| chr19:55377815 -55377887 | KIR2DS4 |
| chr19:55377250 -55377374 | KIR2DS4 |
| chr19:55370524 -55370594 | KIR2DS4 |
| chr19:55367064 -55367377 | KIR2DS4 |
| chr19:55365192 -55365511 | KIR2DS4 |
| chr19:55363443 -55363747 | KIR2DS4 |
| chr19:55362665 -55362720 | KIR2DS4 |
| chr19:55361888 -55361974 | KIR2DS4 |
| chr19:54270960 -54271760 | RETRO-HMGN1 |
| chr19:52327427 -52328227 | FPR3 |
| chr19:51302708 -51303508 | C19ORF48 |
| chr19:4850013 -4850813 | PLIN3 |
| chr19:47892946 -47893746 |  |
| chr19:46411875 -46412675 |  |
| chr19:45296763 -45297563 | CBLC |
| chr19:4416544 -4417344 | CHAF1A |
| chr19:43864407 -43864435 | CD177 |
| chr19:43863290 -43863434 | CD177 |
| chr19:43860134 -43860270 | CD177 |
| chr19:43859803 -43859945 | CD177 |
| chr19:43859657 -43859735 | CD177 |
| chr19:43858349 -43858554 | CD177 |
| chr19:43857995 -43858155 | CD177 |
| chr19:43857815 -43857928 | CD177 |
| chr19:4215338 -4216138 | ANKRD24 |
| chr19:41020596 -41021396 | SPTBN4 |
| chr19:38200424 -38201224 | ZNF607 |
| chr19:37340894 -37341225 | ZNF790 |
| chr19:37329229 -37329296 | ZNF790 |
| chr19:37328930 -37329007 | ZNF790 |
| chr19:37328564 -37328939 | ZNF790 |
| chr19:37316503 -37316604 | ZNF790 |
| chr19:37314559 -37314702 | ZNF790 |
| chr19:37314177 -37314292 | ZNF790 |
| chr19:37309214 -37311026 | ZNF790 |
| chr19:3480748 -3481548 |  |
| chr19:33289675 -33290475 | TDRD12 |
| chr19:32575473 -32576273 |  |
| chr19:2916912 -2918484 | ZNF57 |
| chr19:2916585 -2916627 | ZNF57 |
| chr19:2916474 -2916518 | ZNF57 |
| chr19:2916087 -2916215 | ZNF57 |
| chr19:2916066 -2916257 | ZNF57 |
| chr19:2915706 -2915746 | ZNF57 |
| chr19:2915510 -2915656 | ZNF57 |
| chr19:2912124 -2912269 | ZNF57 |
| chr19:2907096 -2907167 | ZNF57 |
| chr19:2900886 -2901056 | ZNF57 |
| chr19:2768583 -2769383 | SGTA |
| chr19:2417779 -2418579 | TMPRSS9 |
| chr19:24129959 -24130759 |  |
| chr19:23853588 -23854388 | ZNF675 |
| chr19:23667299 -23668099 |  |
| chr19:23181433 -23182233 | ZNF728 |
| chr19:23057711 -23058511 |  |
| chr19:22913107 -22913907 |  |
| chr19:22872936 -22873736 |  |
| chr19:21932987 -21933787 | ZNF100 |
| chr19:21658211 -21659011 |  |
| chr19:21624039 -21624839 |  |
| chr19:21461216 -21462016 |  |
| chr19:20914901 -20915701 |  |
| chr19:20674235 -20675035 |  |
| chr19:20550381 -20551181 | ZNF826P |
| chr19:20339753 -20340553 |  |
| chr19:19939289 -19940089 | RETRO-ISCA1 |
| chr19:1620676 -1621476 | TCF3 |
| chr19:15454611 -15455411 |  |
| chr19:14961202 -14962002 |  |
| chr19:14720866 -14721961 | CLEC17A |
| chr19:14719838 -14719868 | CLEC17A |
| chr19:14717795 -14717924 | CLEC17A |
| chr19:14710833 -14711004 | CLEC17A |
| chr19:14710519 -14710634 | CLEC17A |
| chr19:14707901 -14707983 | CLEC17A |
| chr19:14707678 -14707835 | CLEC17A |
| chr19:14706076 -14706137 | CLEC17A |
| chr19:14705561 -14705622 | CLEC17A |
| chr19:14705434 -14705504 | CLEC17A |
| chr19:14705292 -14705371 | CLEC17A |
| chr19:14703083 -14703180 | CLEC17A |
| chr19:14698416 -14698513 | CLEC17A |
| chr19:14694159 -14694256 | CLEC17A |
| chr19:14693886 -14694025 | CLEC17A |
| chr19:12369539 -12370339 | ZNF44 |
| chr19:10873403 -10874203 | DNM2 |
| chr18:18690769 -18691822 | ROCK1 |
| chr18:18650483 -18650584 | ROCK1 |
| chr18:18629731 -18629851 | ROCK1 |
| chr18:18629043 -18629200 | ROCK1 |
| chr18:18625243 -18625438 | ROCK1 |
| chr18:18624053 -18624157 | ROCK1 |
| chr18:18622516 -18622680 | ROCK1 |
| chr18:18622048 -18622206 | ROCK1 |
| chr18:18619423 -18619534 | ROCK1 |
| chr18:18608727 -18608906 | ROCK1 |
| chr18:18603571 -18603651 | ROCK1 |
| chr18:18600102 -18600210 | ROCK1 |
| chr18:18595382 -18595450 | ROCK1 |
| chr18:18588010 -18588165 | ROCK1 |
| chr18:18586650 -18586761 | ROCK1 |
| chr18:18586302 -18586568 | ROCK1 |
| chr18:18572782 -18572908 | ROCK1 |
| chr18:18571127 -18571297 | ROCK1 |
| chr18:18566901 -18567081 | ROCK1 |
| chr18:18564302 -18564506 | ROCK1 |
| chr18:18562714 -18562803 | ROCK1 |
| chr18:18559861 -18559975 | ROCK1 |
| chr18:18550299 -18550484 | ROCK1 |
| chr18:18549066 -18549179 | ROCK1 |
| chr18:18548723 -18548831 | ROCK1 |
| chr18:18547703 -18547911 | ROCK1 |
| chr18:18546868 -18547047 | ROCK1 |
| chr18:18540087 -18540177 | ROCK1 |
| chr18:18539791 -18539899 | ROCK1 |
| chr18:18535118 -18535216 | ROCK1 |
| chr18:18534734 -18535015 | ROCK1 |
| chr18:18533529 -18533756 | ROCK1 |
| chr18:18529691 -18531358 | ROCK1 |
| chr17:7590685 -7590866 | TP53 |
| chr17:7580633 -7580762 | TP53 |
| chr17:7579690 -7579950 | TP53 |
| chr17:7579302 -7579600 | TP53 |
| chr17:7578361 -7578821 | TP53 |
| chr17:7577834 -7578299 | TP53 |
| chr17:7577489 -7577618 | TP53 |
| chr17:7577009 -7577165 | TP53 |
| chr17:7576843 -7576936 | TP53 |
| chr17:7576515 -7576667 | TP53 |
| chr17:7573917 -7574043 | TP53 |
| chr17:7571710 -7573018 | TP53 |
| chr17:7565247 -7565342 | TP53 |
| chr17:6616541 -6616750 | SLC13A5 |
| chr17:6610337 -6610485 | SLC13A5 |
| chr17:6609951 -6610107 | SLC13A5 |
| chr17:6607187 -6607385 | SLC13A5 |
| chr17:6606279 -6606467 | SLC13A5 |
| chr17:6604313 -6604455 | SLC13A5 |
| chr17:6599045 -6599222 | SLC13A5 |
| chr17:6599035 -6599270 | SLC13A5 |
| chr17:6598990 -6599019 | SLC13A5 |
| chr17:6597406 -6597526 | SLC13A5 |
| chr17:6596353 -6596491 | SLC13A5 |
| chr17:6594088 -6594269 | SLC13A5 |
| chr17:6590838 -6590995 | SLC13A5 |
| chr17:6588022 -6589667 | SLC13A5 |
| chr17:41835880 -41836166 | SOST |
| chr17:41831093 -41833141 | SOST |
| chr17:37883932 -37884925 | ERBB2 |
| chr17:37883538 -37883810 | ERBB2 |
| chr17:37883058 -37883266 | ERBB2 |
| chr17:37882805 -37882922 | ERBB2 |
| chr17:37881950 -37882116 | ERBB2 |
| chr17:37881570 -37881665 | ERBB2 |
| chr17:37881292 -37881467 | ERBB2 |
| chr17:37880969 -37881174 | ERBB2 |
| chr17:37880155 -37880273 | ERBB2 |
| chr17:37879781 -37879923 | ERBB2 |
| chr17:37879562 -37879720 | ERBB2 |
| chr17:37876030 -37876097 | ERBB2 |
| chr17:37873563 -37873743 | ERBB2 |
| chr17:37872758 -37872868 | ERBB2 |
| chr17:37872544 -37872696 | ERBB2 |
| chr17:37871983 -37872202 | ERBB2 |
| chr17:37871689 -37871799 | ERBB2 |
| chr17:37871529 -37871622 | ERBB2 |
| chr17:37868565 -37868711 | ERBB2 |
| chr17:37868171 -37868310 | ERBB2 |
| chr17:37866583 -37866744 | ERBB2 |
| chr17:37866329 -37866464 | ERBB2 |
| chr17:37866056 -37866144 | ERBB2 |
| chr17:37865561 -37865715 | ERBB2 |
| chr17:37864564 -37864797 | ERBB2 |
| chr17:37863233 -37863404 | ERBB2 |
| chr17:37856244 -37856574 | ERBB2 |
| chr17:37851176 -37851444 | ERBB2 |
| chr17:37844939 -37845063 | ERBB2 |
| chr17:37844383 -37844541 | ERBB2 |
| chr17:3194919 -3195904 | OR3A1 |
| chr17:29705896 -29705959 | NF1 |
| chr17:29701021 -29701705 | NF1 |
| chr17:29694233 -29694306 | NF1 |
| chr17:29687495 -29687984 | NF1 |
| chr17:29685977 -29686043 | NF1 |
| chr17:29685488 -29685650 | NF1 |
| chr17:29684277 -29684397 | NF1 |
| chr17:29683968 -29684118 | NF1 |
| chr17:29683468 -29683610 | NF1 |
| chr17:29679265 -29679442 | NF1 |
| chr17:29677834 -29677868 | NF1 |
| chr17:29677473 -29677496 | NF1 |
| chr17:29677191 -29677346 | NF1 |
| chr17:29676128 -29676279 | NF1 |
| chr17:29670017 -29670163 | NF1 |
| chr17:29667513 -29667673 | NF1 |
| chr17:29665712 -29665833 | NF1 |
| chr17:29665010 -29665167 | NF1 |
| chr17:29664800 -29664908 | NF1 |
| chr17:29664376 -29664610 | NF1 |
| chr17:29663643 -29663942 | NF1 |
| chr17:29663341 -29663506 | NF1 |
| chr17:29661846 -29662059 | NF1 |
| chr17:29661672 -29661758 | NF1 |
| chr17:29657304 -29658932 | NF1 |
| chr17:29654507 -29654867 | NF1 |
| chr17:29652828 -29653280 | NF1 |
| chr17:29592237 -29592367 | NF1 |
| chr17:29588719 -29588885 | NF1 |
| chr17:29587377 -29587543 | NF1 |
| chr17:29586040 -29586157 | NF1 |
| chr17:29585352 -29585530 | NF1 |
| chr17:29584695 -29584754 | NF1 |
| chr17:29579946 -29580028 | NF1 |
| chr17:29576963 -29577832 | NF1 |
| chr17:29575992 -29576147 | NF1 |
| chr17:29562926 -29563049 | NF1 |
| chr17:29562619 -29562800 | NF1 |
| chr17:29560010 -29560241 | NF1 |
| chr17:29559708 -29559909 | NF1 |
| chr17:29559081 -29559217 | NF1 |
| chr17:29557850 -29557953 | NF1 |
| chr17:29557268 -29557410 | NF1 |
| chr17:29556033 -29557002 | NF1 |
| chr17:29554531 -29554634 | NF1 |
| chr17:29554226 -29554319 | NF1 |
| chr17:29553443 -29553712 | NF1 |
| chr17:29552091 -29552278 | NF1 |
| chr17:29550452 -29550595 | NF1 |
| chr17:29548858 -29549792 | NF1 |
| chr17:29546013 -29546387 | NF1 |
| chr17:29541459 -29541613 | NF1 |
| chr17:29533248 -29533399 | NF1 |
| chr17:29528419 -29528513 | NF1 |
| chr17:29528045 -29528187 | NF1 |
| chr17:29527430 -29527623 | NF1 |
| chr17:29509516 -29510055 | NF1 |
| chr17:29508718 -29508813 | NF1 |
| chr17:29508430 -29508517 | NF1 |
| chr17:29496899 -29497025 | NF1 |
| chr17:29490194 -29490404 | NF1 |
| chr17:29486018 -29486121 | NF1 |
| chr17:29482991 -29483154 | NF1 |
| chr17:29421935 -29422397 | NF1 |
| chr16:57625516 -57625603 | GPR114 |
| chr16:57624514 -57624692 | GPR114 |
| chr16:57609340 -57611117 | GPR114 |
| chr16:57608717 -57609014 | GPR114 |
| chr16:57604320 -57604457 | GPR114 |
| chr16:57601758 -57602046 | GPR114 |
| chr16:57601372 -57601513 | GPR114 |
| chr16:57600501 -57600673 | GPR114 |
| chr16:57598936 -57599072 | GPR114 |
| chr16:57597750 -57597901 | GPR114 |
| chr16:57596993 -57597169 | GPR114 |
| chr16:57596286 -57596381 | GPR114 |
| chr16:57595958 -57596079 | GPR114 |
| chr16:57576591 -57577095 | GPR114 |
| chr16:57576560 -57576623 | GPR114 |
| chr16:2998679 -3001218 | FLYWCH1 |
| chr16:2990023 -2990103 | FLYWCH1 |
| chr16:2988175 -2988467 | FLYWCH1 |
| chr16:2986520 -2987395 | FLYWCH1 |
| chr16:2986323 -2986436 | FLYWCH1 |
| chr16:2984606 -2984678 | FLYWCH1 |
| chr16:2983707 -2983990 | FLYWCH1 |
| chr16:2983121 -2983593 | FLYWCH1 |
| chr16:2980401 -2980891 | FLYWCH1 |
| chr16:2979604 -2980021 | FLYWCH1 |
| chr16:2964167 -2964300 | FLYWCH1 |
| chr16:2961970 -2962165 | FLYWCH1 |
| chr15:90645498 -90645746 | IDH2 |
| chr15:90634775 -90634886 | IDH2 |
| chr15:90633701 -90633886 | IDH2 |
| chr15:90631809 -90631989 | IDH2 |
| chr15:90631581 -90631744 | IDH2 |
| chr15:90630661 -90630817 | IDH2 |
| chr15:90630334 -90630505 | IDH2 |
| chr15:90628497 -90628629 | IDH2 |
| chr15:90628223 -90628340 | IDH2 |
| chr15:90628038 -90628150 | IDH2 |
| chr15:90626267 -90627595 | IDH2 |
| chr15:75341481 -75343077 | PPCDC |
| chr15:75340884 -75341072 | PPCDC |
| chr15:75336717 -75336865 | PPCDC |
| chr15:75335772 -75335887 | PPCDC |
| chr15:75320578 -75320804 | PPCDC |
| chr15:75315886 -75315977 | PPCDC |
| chr15:52011595 -52013238 | SCG3 |
| chr15:52005520 -52005620 | SCG3 |
| chr15:51993294 -51993451 | SCG3 |
| chr15:51991506 -51991609 | SCG3 |
| chr15:51988062 -51988198 | SCG3 |
| chr15:51984346 -51984543 | SCG3 |
| chr15:51981406 -51981575 | SCG3 |
| chr15:51980447 -51980609 | SCG3 |
| chr15:51975406 -51975641 | SCG3 |
| chr15:51975266 -51975331 | SCG3 |
| chr15:51974704 -51974776 | SCG3 |
| chr15:51973540 -51974044 | SCG3 |
| chr14:74061743 -74062478 | ACOT4 |
| chr14:74060396 -74060618 | ACOT4 |
| chr14:74058400 -74059130 | ACOT4 |
| chr14:32623817 -32628944 | ARHGAP5 |
| chr14:32621619 -32621744 | ARHGAP5 |
| chr14:32619098 -32619249 | ARHGAP5 |
| chr14:32615459 -32615556 | ARHGAP5 |
| chr14:32586336 -32586503 | ARHGAP5 |
| chr14:32563299 -32563432 | ARHGAP5 |
| chr14:32559698 -32563602 | ARHGAP5 |
| chr14:32546485 -32546651 | ARHGAP5 |
| chr13:49054124 -49055132 | RB1 |
| chr13:49051481 -49051550 | RB1 |
| chr13:49050573 -49050989 | RB1 |
| chr13:49050526 -49050668 | RB1 |
| chr13:49047486 -49047536 | RB1 |
| chr13:49039331 -49039514 | RB1 |
| chr13:49039124 -49039257 | RB1 |
| chr13:49037857 -49037981 | RB1 |
| chr13:49033814 -49033979 | RB1 |
| chr13:49030330 -49030495 | RB1 |
| chr13:49027119 -49027257 | RB1 |
| chr13:49018954 -49019367 | RB1 |
| chr13:48955373 -48955589 | RB1 |
| chr13:48954291 -48954387 | RB1 |
| chr13:48954179 -48954230 | RB1 |
| chr13:48953720 -48953796 | RB1 |
| chr13:48951044 -48951180 | RB1 |
| chr13:48947531 -48947638 | RB1 |
| chr13:48942653 -48942750 | RB1 |
| chr13:48941620 -48941749 | RB1 |
| chr13:48939020 -48939117 | RB1 |
| chr13:48936941 -48937103 | RB1 |
| chr13:48934143 -48934883 | RB1 |
| chr13:48923082 -48923169 | RB1 |
| chr13:48921951 -48922009 | RB1 |
| chr13:48919206 -48919345 | RB1 |
| chr13:48916725 -48916860 | RB1 |
| chr13:48881406 -48881552 | RB1 |
| chr13:48877877 -48878195 | RB1 |
| chr13:114117045 -114117413 | DCUN1D2 |
| chr13:114115359 -114115461 | DCUN1D2 |
| chr13:114113617 -114113868 | DCUN1D2 |
| chr13:114110124 -114112433 | DCUN1D2 |
| chr13:113192973-113193278 | DCUN1D2 |
| chr13:113192739-113192934 | DCUN1D2 |
| chr13:113191396-113191579 | DCUN1D2 |
| chr13:113186146-113186522 | DCUN1D2 |
| chr13:113182772-113183069 | DCUN1D2 |
| chr13:113176430-113176580 | DCUN1D2 |
| chr12:96184023 -96184546 | NTN4 |
| chr12:96180707 -96181256 | NTN4 |
| chr12:96131634 -96131932 | NTN4 |
| chr12:96106980 -96107126 | NTN4 |
| chr12:96104209 -96104417 | NTN4 |
| chr12:96077264 -96077497 | NTN4 |
| chr12:96076473 -96076608 | NTN4 |
| chr12:96063844 -96063932 | NTN4 |
| chr12:96059576 -96059766 | NTN4 |
| chr12:96051574 -96053008 | NTN4 |
| chr12:69233044 -69234224 | MDM2 |
| chr12:69230442 -69230539 | MDM2 |
| chr12:69229599 -69229774 | MDM2 |
| chr12:69222541 -69222721 | MDM2 |
| chr12:69218325 -69218441 | MDM2 |
| chr12:69218133 -69218220 | MDM2 |
| chr12:69214095 -69214204 | MDM2 |
| chr12:69210582 -69210808 | MDM2 |
| chr12:69209398 -69209510 | MDM2 |
| chr12:69208373 -69208479 | MDM2 |
| chr12:69207324 -69207418 | MDM2 |
| chr12:69202978 -69203082 | MDM2 |
| chr12:69202787 -69202876 | MDM2 |
| chr12:69201946 -69202281 | MDM2 |
| chr12:58145948 -58146174 | CDK4 |
| chr12:58145273 -58145529 | CDK4 |
| chr12:58144980 -58145135 | CDK4 |
| chr12:58144696 -58144883 | CDK4 |
| chr12:58144429 -58144558 | CDK4 |
| chr12:58143227 -58143297 | CDK4 |
| chr12:58142955 -58143110 | CDK4 |
| chr12:58141995 -58142410 | CDK4 |
| chr12:57864090 -57866055 | GLI1 |
| chr12:57863204 -57863491 | GLI1 |
| chr12:57861767 -57862017 | GLI1 |
| chr12:57861106 -57861290 | GLI1 |
| chr12:57860013 -57860182 | GLI1 |
| chr12:57859561 -57859934 | GLI1 |
| chr12:57859148 -57859489 | GLI1 |
| chr12:57858884 -57859048 | GLI1 |
| chr12:57858446 -57858736 | GLI1 |
| chr12:57857772 -57857884 | GLI1 |
| chr12:57857438 -57857584 | GLI1 |
| chr12:57854264 -57854348 | GLI1 |
| chr12:57853908 -57853994 | GLI1 |
| chr12:25403675 -25403864 | KRAS |
| chr12:25398198 -25398339 | KRAS |
| chr12:25380158 -25380356 | KRAS |
| chr12:25378538 -25378717 | KRAS |
| chr12:25368361 -25368504 | KRAS |
| chr12:25362172 -25362855 | KRAS |
| chr12:21621430 -21624186 | PYROXD1 |
| chr12:21620405 -21620562 | PYROXD1 |
| chr12:21615664 -21615806 | PYROXD1 |
| chr12:21614932 -21615064 | PYROXD1 |
| chr12:21613949 -21614098 | PYROXD1 |
| chr12:21608919 -21609039 | PYROXD1 |
| chr12:21608056 -21608236 | PYROXD1 |
| chr12:21605005 -21605098 | PYROXD1 |
| chr12:21602487 -21602635 | PYROXD1 |
| chr12:21598271 -21598410 | PYROXD1 |
| chr12:21593292 -21593392 | PYROXD1 |
| chr12:21590528 -21590758 | PYROXD1 |
| chr11:55563022 -55563986 | OR5D14 |
| chr11:536843 -537297 | HRAS |
| chr11:535406 -535560 | HRAS |
| chr11:535247 -535349 | HRAS |
| chr11:534508 -534597 | HRAS |
| chr11:534202 -534385 | HRAS |
| chr11:533756 -533986 | HRAS |
| chr11:533443 -533622 | HRAS |
| chr11:533267 -533368 | HRAS |
| chr11:532232 -532765 | HRAS |
| chr11:102576334 -102576478 | MMP27 |
| chr11:102575258 -102575516 | MMP27 |
| chr11:102573691 -102573859 | MMP27 |
| chr11:102573474 -102573622 | MMP27 |
| chr11:102567395 -102567576 | MMP27 |
| chr11:102567092 -102567232 | MMP27 |
| chr11:102565688 -102565838 | MMP27 |
| chr11:102564627 -102564806 | MMP27 |
| chr11:102563659 -102563782 | MMP27 |
| chr11:102562405 -102562751 | MMP27 |
| chr10:89725034 -89725697 | PTEN |
| chr10:89720641 -89721076 | PTEN |
| chr10:89717600 -89717786 | PTEN |
| chr10:89711865 -89712026 | PTEN |
| chr10:89692760 -89693254 | PTEN |
| chr10:89690793 -89690856 | PTEN |
| chr10:89685260 -89685324 | PTEN |
| chr10:89653772 -89654093 | PTEN |
| chr10:89626420 -89626816 | PTEN |
| chr10:89622860 -89624315 | PTEN |
| chr10:26589707 -26593497 | GAD2 |
| chr10:26581821 -26581930 | GAD2 |
| chr10:26581384 -26581511 | GAD2 |
| chr10:26575264 -26575433 | GAD2 |
| chr10:26569928 -26570026 | GAD2 |
| chr10:26562555 -26562639 | GAD2 |
| chr10:26559559 -26559695 | GAD2 |
| chr10:26558038 -26558112 | GAD2 |
| chr10:26534840 -26534939 | GAD2 |
| chr10:26518581 -26518716 | GAD2 |
| chr10:26513458 -26513699 | GAD2 |
| chr10:26512806 -26512916 | GAD2 |
| chr10:26507962 -26508259 | GAD2 |
| chr10:26507112 -26507264 | GAD2 |
| chr10:26506761 -26506930 | GAD2 |
| chr10:26506529 -26506608 | GAD2 |
| chr10:26505226 -26505824 | GAD2 |
| chr10:13141496 -13141662 | CCDC3 |
| chr10:13141074 -13141319 | CCDC3 |
| chr10:13140515 -13140612 | CCDC3 |
| chr10:13115858 -13116110 | CCDC3 |
| chr10:13091664 -13091951 | CCDC3 |
| chr10:13043187 -13043707 | CCDC3 |
| chr10:13040328 -13040522 | CCDC3 |
| chr10:12938617 -12940689 | CCDC3 |
| chr1:99043875 -99044675 |  |
| chr1:97639059 -97639859 | DPYD |
| chr1:9074450 -9075250 | SLCA7 |
| chr1:90718432 -90719232 |  |
| chr1:89664468 -89664625 | GBP4 |
| chr1:89662783 -89662997 | GBP4 |
| chr1:89660970 -89661117 | GBP4 |
| chr1:89658976 -89659105 | GBP4 |
| chr1:89658577 -89658793 | GBP4 |
| chr1:89656934 -89657199 | GBP4 |
| chr1:89655711 -89656011 | GBP4 |
| chr1:89654255 -89654487 | GBP4 |
| chr1:89652673 -89652795 | GBP4 |
| chr1:89652006 -89652219 | GBP4 |
| chr1:89650910 -89651091 | GBP4 |
| chr1:89648171 -89648220 | GBP4 |
| chr1:89647446 -89648071 | GBP4 |
| chr1:89646821 -89651162 | GBP4 |
| chr1:88421296 -88422096 |  |
| chr1:88123296 -88124096 |  |
| chr1:8272702 -8273502 |  |
| chr1:82087267 -82088067 | LPHN2 |
| chr1:79940806 -79941606 |  |
| chr1:763176 -763976 | LOC643837 |
| chr1:75923451 -75924251 | SLC44AB |
| chr1:7482344 -7483144 | CAMTA1 |
| chr1:6919494 -6920294 | CAMTA1 |
| chr1:63512604 -63513404 |  |
| chr1:5655843 -5656643 |  |
| chr1:5651508 -5652308 |  |
| chr1:55644513 -55645313 | USP24 |
| chr1:51618974 -51619774 |  |
| chr1:50607201 -50608001 | ELAVL4 |
| chr1:49813689 -49814489 | AGBL4 |
| chr1:49813318 -49814118 | AGBL4 |
| chr1:48425822 -48426622 | TRABD2B |
| chr1:47341763 -47342563 | CYP422F |
| chr1:45416216 -45417016 | EIF2B3 |
| chr1:45310486 -45311286 |  |
| chr1:41765158 -41765958 |  |
| chr1:4026171 -4026971 |  |
| chr1:3397002 -3397687 | ARHGEF16 |
| chr1:3396366 -3396487 | ARHGEF16 |
| chr1:3396071 -3396164 | ARHGEF16 |
| chr1:3394429 -3395186 | ARHGEF16 |
| chr1:3392524 -3392636 | ARHGEF16 |
| chr1:3391262 -3391356 | ARHGEF16 |
| chr1:3389947 -3391031 | ARHGEF16 |
| chr1:3389323 -3389804 | ARHGEF16 |
| chr1:3388161 -3388365 | ARHGEF16 |
| chr1:3385987 -3386167 | ARHGEF16 |
| chr1:3385098 -3385510 | ARHGEF16 |
| chr1:3383525 -3383911 | ARHGEF16 |
| chr1:3382559 -3382767 | ARHGEF16 |
| chr1:3379620 -3380246 | ARHGEF16 |
| chr1:3370980 -3371385 | ARHGEF16 |
| chr1:32657721 -32658521 | TXLNA |
| chr1:30070624 -30071424 |  |
| chr1:28560466 -28561266 |  |
| chr1:28019285 -28020085 |  |
| chr1:28019265 -28020065 |  |
| chr1:25756421 -25756693 | RHCE HG18 |
| chr1:25755434 -25755550 | RHCE HG18 |
| chr1:25747120 -25747442 | RHCE HG18 |
| chr1:25746236 -25746299 | RHCE HG18 |
| chr1:25737821 -25737979 | RHCE HG18 |
| chr1:25735164 -25735370 | RHCE HG18 |
| chr1:25729077 -25729247 | RHCE HG18 |
| chr1:25718475 -25718642 | RHCE HG18 |
| chr1:25717230 -25717416 | RHCE HG18 |
| chr1:25715457 -25715614 | RHCE HG18 |
| chr1:25712192 -25712316 | RHCE HG18 |
| chr1:25711979 -25712345 | RHCE HG18 |
| chr1:25701830 -25701929 | RHCE HG18 |
| chr1:25696948 -25697041 | RHCE HG18 |
| chr1:25688731 -25689054 | RHCE HG18 |
| chr1:24921137 -24921937 | NCMAP |
| chr1:246946455 -246947255 | LOC149134 |
| chr1:245775866 -245776666 | KIF26B |
| chr1:244217080 -244220788 | ZNF238 |
| chr1:244214551 -244214756 | ZNF238 |
| chr1:23545867 -23546667 |  |
| chr1:231622671 -231623471 |  |
| chr1:228174026 -228174826 |  |
| chr1:228173963 -228174763 |  |
| chr1:225747394 -225748194 | ENAH |
| chr1:225747278 -225748078 | ENAH |
| chr1:225747263 -225748063 | ENAH |
| chr1:225747259 -225748059 | ENAH |
| chr1:223451522 -223452322 | SUSD4 |
| chr1:222721822 -222722622 |  |
| chr1:217801363 -217802163 | GPATCH2 |
| chr1:214846320 -214847120 |  |
| chr1:214203627 -214204427 | PROX1 |
| chr1:20662280 -20663080 | VWA5B1 |
| chr1:199960242 -199961042 |  |
| chr1:193453507 -193454307 |  |
| chr1:190668627 -190669427 | LOC440704 |
| chr1:190170988 -190171788 | FAM5C |
| chr1:185458185 -185458985 |  |
| chr1:180068768 -180069568 | CEP350 |
| chr1:17698801 -17699601 | PAD16 |
| chr1:169322230 -169323030 | NME7 |
| chr1:16815664 -16816464 | CROCCP3 |
| chr1:163717097 -163717897 |  |
| chr1:158177951 -158178751 |  |
| chr1:157614966 -157615766 |  |
| chr1:156994233 -156995033 | ARHGEF11 |
| chr1:155974080 -155974880 |  |
| chr1:153320994 -153321326 | PGLYRP4 |
| chr1:153320350 -153320464 | PGLYRP4 |
| chr1:153318568 -153318677 | PGLYRP4 |
| chr1:153317635 -153317868 | PGLYRP4 |
| chr1:153317491 -153317856 | PGLYRP4 |
| chr1:153315556 -153315694 | PGLYRP4 |
| chr1:153314093 -153314265 | PGLYRP4 |
| chr1:153312847 -153313065 | PGLYRP4 |
| chr1:153309647 -153309785 | PGLYRP4 |
| chr1:153302586 -153303431 | PGLYRP4 |
| chr1:152024788 -152025588 |  |
| chr1:144947282 -144948082 | LOC100131643 |
| chr1:143301814 -143302614 |  |
| chr1:142300616 -142301416 |  |
| chr1:141841694 -141842494 |  |
| chr1:13766902 -13767702 |  |
| chr1:118576699 -118577499 | SPAG17 |
| chr1:117210608 -117211408 |  |
| chr1:116362346 -116363146 |  |
| chr1:115259269 -115259525 | NRAS |
| chr1:115258661 -115258808 | NRAS |
| chr1:115256411 -115256609 | NRAS |
| chr1:115252180 -115252359 | NRAS |
| chr1:115251142 -115251285 | NRAS |
| chr1:115250765 -115250823 | NRAS |
| chr1:115137235 -115138035 | DENN2DC |
| chr1:113942782 -113943582 | MAGI3 |
| chr1:11356237 -11357037 |  |
| chr1:113158858 -113159658 | ST7L |
| chr1:109012020 -109012820 | NBFF6 |
| chr1:108686925 -108687725 | SLC25A24 |
| chr1:104943971 -104944771 |  |
