# Supplementary material for: Tumor-specific mutations in low-frequency genes affect their functional properties
Source: J Neurooncol. 2015 Feb 19;122(3):461–70. doi: 10.1007/s11060-015-1741-1 (PMC4436689; doi:10.1007/s11060-015-1741-1)
Supplement: Supplementary file 9 — Supplementary material 9 (DOC 109 kb) [file 11060_2015_1741_MOESM9_ESM.doc]

Supplementary table 4. Targeted resequencing

Abbreviations; LOH: loss of heterozygosity; TRS: targeted resequencing
